# Tubular Nanostructures from Large‐Pore 2D Covalent Organic Frameworks

**DOI:** 10.1002/anie.202505935

**Published:** 2025-04-04

**Authors:** Joaquín Almarza, Ian Cardillo‐Zallo, Karol Strutyński, Marta Martínez‐Abadía, Natalia M. Padial, Carlos Martí‐Gastaldo, Manuel Melle‐Franco, Andrei N. Khlobystov, Aurelio Mateo‐Alonso

**Affiliations:** ^1^ POLYMAT University of the Basque Country UPV/EHU Avenida de Tolosa 72 Donostia‐San Sebastián 20018 Spain; ^2^ School of Chemistry University of Nottingham University Park Nottingham NG7 2RD UK; ^3^ The Nanoscale and Microscale Research Centre University of Nottingham University Park Nottingham NG7 2RD UK; ^4^ CICECO – Aveiro Institute of Materials, Department of Chemistry University of Aveiro Aveiro 3810‐193 Portugal; ^5^ Instituto de Ciencia Molecular Universidad de Valencia Paterna 46980 Spain; ^6^ Ikerbasque, Basque Foundation for Science Bilbao 48009 Spain

**Keywords:** Covalent organic frameworks, Nanotubes, Mesoporous, Large‐pore, Polycylic aromatic hydrocarbons

## Abstract

The synthesis of a wavy mesoporous 2D covalent organic framework (COF) with a 6‐nm hexagonal pore lattice (**Joa‐COF‐1**) is reported. This has been achieved by the synthesis of a terpyrenyl linker of approximately 2.7 nm in length and its subsequent condensation with a 3‐connected non‐planar cata‐hexabenzocoronene. **Joa‐COF‐1** exists as non‐covalent tubular domains composed of π‐stacked 4 to 5 pores in 2D COF sections that can be separated by mild sonication, resulting in a family of tubular COF nanostructures that combine a 1D morphology with accessible mesopores.

## Graphical abstract

A wavy mesoporous 2D covalent organic framework (COF) has been synthesized with a 6‐nm hexagonal pore lattice. This COF forms non‐covalent tubular domains that can be separated by mild sonication, yielding a family of tubular COF nanostructures that combine a 1D morphology with accessible mesopores.

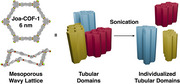

Nanotubes are tubular nanostructures with diameters on the nanometer scale characterized by their hollow interior. This internal cavity provides them with a low density and with the ability to host something and transport charges,^[^ ^1^ ^]^ ions,^[^ ^2^, ^3^ ^]^ water.^[^ ^3^ ^]^ Carbon nanotubes are the most representative examples of these tubular nanostructures,^[^ ^4^ ^]^ yet nanotubes can also be crafted through the supramolecular^[^ ^5^, ^6^, ^7^, ^8^, ^9^, ^10^ ^]^ and covalent^[^ ^11^, ^12^ ^]^ linkage of organic molecules, allowing precise control over their diameter. However, despite being open‐pore structures, supramolecular and covalent nanotubes rarely show porosity.^[^ ^11^ ^]^

In contrast, 2D covalent organic frameworks (COFs) are highly porous crystalline polymers.^[^ ^13^, ^14^, ^15^, ^16^, ^17^, ^18^, ^19^ ^]^ The size and shape of 2D COFs’ pores are determined by the geometry and size of the monomers, and therefore, mesoporous 2D COFs can be obtained by a judicious design and selection of monomers of nanometer dimensions.^[^ ^20^ ^]^ The advantage of large mesopores is that they allow the diffusion of large molecules into the 2D COFs, such as polymers^[^ ^21^, ^22^, ^23^ ^]^ and biomolecules,^[^ ^24^, ^25^, ^26^ ^]^ providing new application potential for 2D COFs.^[^ ^20^, ^25^, ^27^ ^]^ However, the crystallinity and structural stability of large‐pore 2D COFs are challenged during synthesis and activation, respectively, due to the limited solubility of large organic monomers and the limited interlayer interactions per unit area as the pore size increases.

We have recently introduced the use of non‐planar cata‐hexabenzocoronene (HBC) as nodes in 2D and 3D COFs.^[^ ^28^, ^29^, ^30^ ^]^ HBC adopts a twisted and rigid structure as the result of steric congestion between the hydrogens in the peripheral benzene rings, which squeeze such rings above and below the basal plane in an alternated fashion, giving rise to a double bowl structure (Figure 1). The condensation of three‐connected non‐planar HBC nodes with linear diboronic ester linkers generates a wavy honeycomb 2D lattice with concave‐convex complementarity that guides the stacking of 2D COF layers.^[^ ^28^, ^29^ ^]^ We hypothesize that this complementarity would compensate for limited interlayer interactions per unit of area as the pore size grows, enabling the synthesis of isoreticular mesoporous 2D COFs. To achieve this, we have designed and synthesized a terpyrenyl diboronic acid linker of approximately 2.7 nm in length (terpyrenyl **1**, Figure 1). The condensation of terpyrenyl **1** with a 3‐connected hexahydroxy‐cata‐hexabenzocoronene (HBC **2**), generates a 2D COF with 6‐nm hexagonal pore lattice (**Joa‐COF‐1**), which is among the 2D COFs with the largest mesopores.

**Figure 1:**
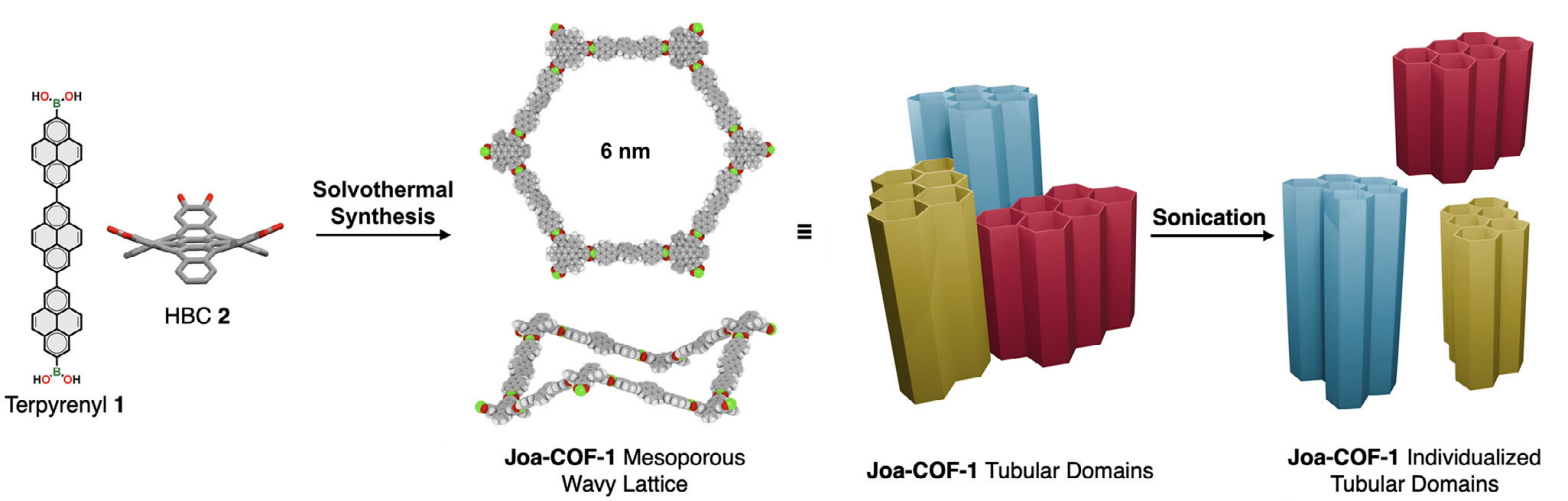
Schematic representation of the synthesis of **Joa‐COF‐1** from terpyrenyl **1** and HBC **2**, highlighting its domain separation into individualized tubular 2D COF nanostructures.

To our surprise, **Joa‐COF‐1** is comprised of non‐covalent, tubular domains of π‐stacked 2D COF sections, each containing 4 to 5 pores. These domains can be separated by mild sonication, resulting in 2D COF tubular nanostructures, as observed through high‐resolution transmission electron microscopy (HR‐TEM). While COF exfoliation along the plane^[^ ^19^, ^31^, ^32^, ^33^ ^]^ and the synthesis of supramolecular and covalent nanotubes^[^ ^9^, ^11^, ^12^, ^34^, ^35^ ^]^ have been previously documented, the disassembly of COF crystallites along the channel into tubular nanostructures is, to the best of our knowledge, an unprecedented phenomenon. This results in a hybrid family of 2D COF tubular nanostructures that combine a 1D morphology with accessible mesopores.

The synthesis of **Joa‐COF‐1** involves the synthesis of terpyrenyl **1** and HBC **2**. Terpyrenyl **1** was synthesized in four steps from pyrene (Scheme 1). First, two bispinacol boronic esters were introduced in the 2,7 positions of pyrene through iridium catalyzed borylation to obtain diboronic ester **3**, which was subsequently transformed into 2,7‐dibromopyrene (**4**) by bromination following a reported method.^[^ ^36^ ^]^ Then 2,7‐dibromopyrene (**4**) was allowed to react with an excess of pyrene diboronic ester **3**, though Suzuki coupling, to obtain the corresponding terpyrenyl diboronic ester **5**, which was subsequently hydrolyzed into the desired terpyrenyl diboronic acid **1**. Despite the three covalently‐linked pyrenes and the two terminal boronic acids, terpyrenyl **1** was still soluble in polar solvents such as DMSO, DMF, *N*,*N*’‐dimethyl acetamide, and 1,4‐dioxane at room temperature. This level of solubility enabled the characterization of terpyrenyl **1** by ^1^H and ^13^C NMR and by MALDI‐TOF mass spectrometry. HBC **2** was synthesized following a reported procedure.^[^ ^28^ ^]^

**Scheme 1:**
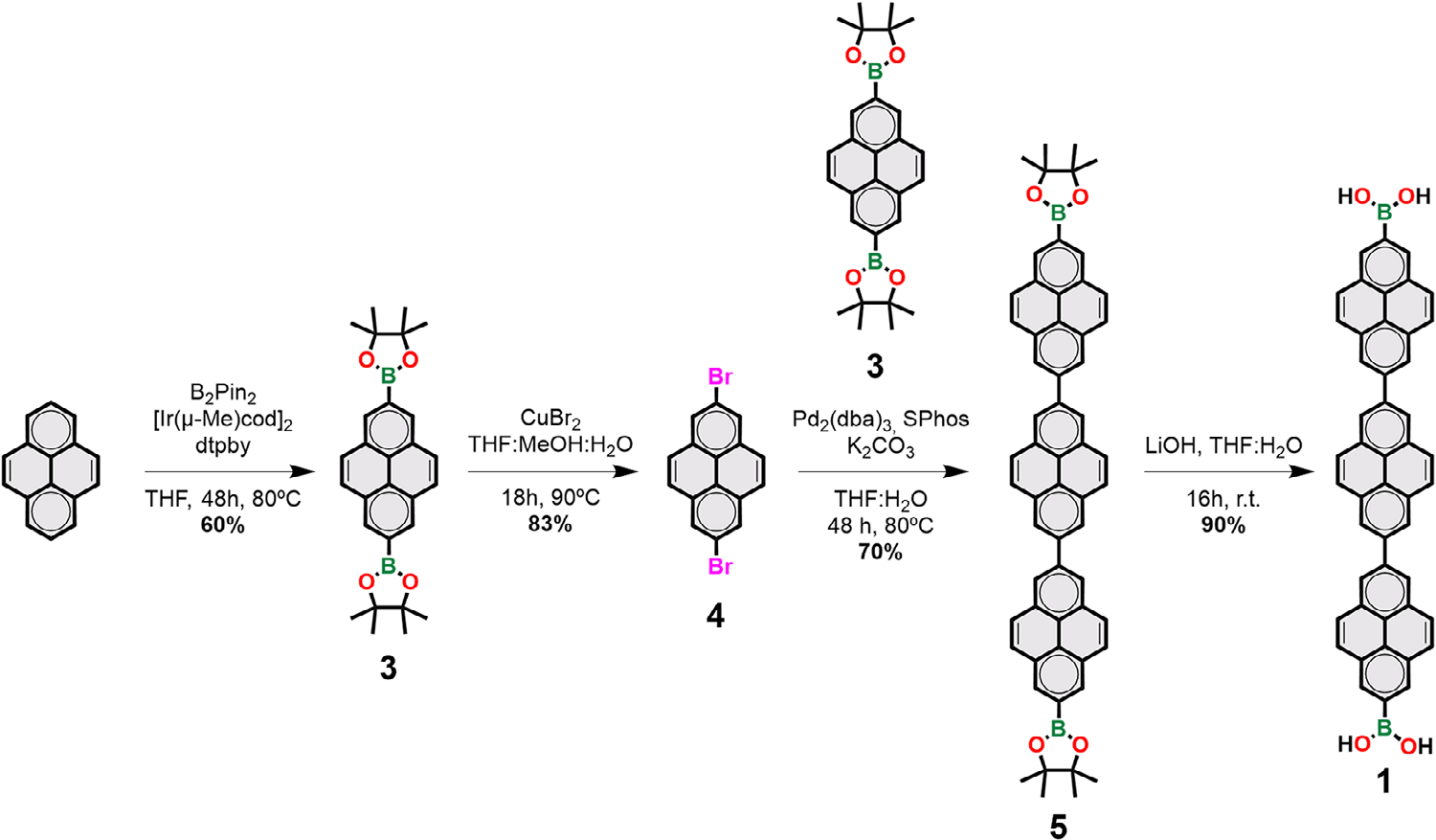
Synthesis of terpyrenyl **1**.

**Joa‐COF‐1** was obtained as a greenish solid (92% yield) by solvothermal condensation of terpyrenyl **1** and HBC **2** in a mixture of 1,4‐dioxane and mesitylene 2:1, v:v, at 120 °C for 72 h (Figure 2a). Similarly to other 2D COFs with non‐planar HBC nodes reported by some of us,^[^ ^28^, ^29^ ^]^ **Joa‐COF‐1** is intrinsically wavy as a consequence of the twisted and rigid structure of the HBC nodes (Figure 2b,c), where the alternated arrangement of HBC nodes with catechols pointing above and below the plane generates a chair‐like honeycomb lattice. The Fourier‐transform infrared (FT‐IR) spectrum of **Joa‐COF‐1** is consistent with the successful polymerization process of the starting materials as it shows the distinctive C─O stretching bands at 1352 and 1317 cm^−1^ for boronate ester five‐membered rings and the attenuation of O─H band of the catechol and the boronic acid groups (Figure S1). Similarly, solid‐state cross‐polymerization magic angle spinning (CP/MAS) ^1^H and ^13^C NMR spectra show aromatic signals of the HBC and terpyrenyl moieties consistent with the structure of **Joa‐COF‐1** (Figure S2). Thermal gravimetric analysis of **Joa‐COF‐1** reveals that 90% of its mass is retained up to 500 °C under nitrogen (Figure S3). **Joa‐COF‐1** exhibits the typical chemical stability of boronate ester COFs, showing signs of hydrolysis after seven days under ambient conditions. After synthesis, the samples were stored in a glove box to prevent decomposition, where they have remained stable for over a year.

**Figure 2:**
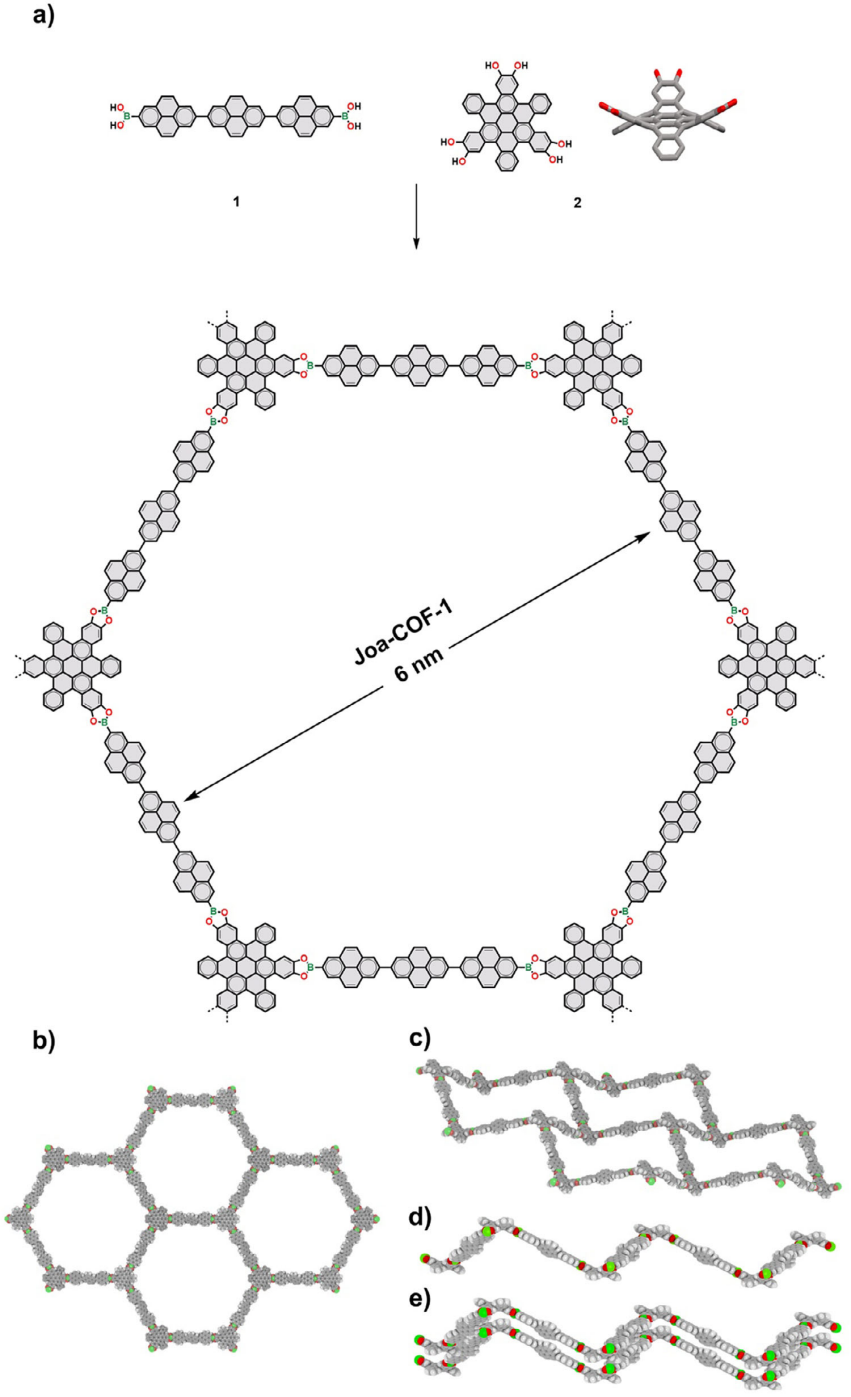
a) Synthesis of **Joa‐COF‐1**. b) Front and c), d) side views of a monolayer of **Joa‐COF‐1**. e) The concave‐convex complementarity between the wavy layers of **Joa‐COF‐1** guides the AA packing.

HR‐TEM imaging was performed using a single‐electron detector camera that allows imaging at a very low flux of the electron beam on the sample of 200–1000 e/(s·nm^2^) (details in Supporting Information). Imaging of **Joa‐COF‐1** particles deposited onto a TEM grid in a dry form revealed a disordered structure with local crystalline order (Figures 3a,b, and S4–S20). Nanocrystalline domains of ∼50 nm can be recognized by parallel lines (d‐spacing of the COF lattice) in the microparticles (Figures 3a,b). EDX analysis indicates the presence of C, O, and B (the latter is overlapping with C peak) (Figure 3c) in agreement with the structure of **Joa‐COF‐1**. This is consistent with X‐ray photoelectron spectroscopy and elemental analysis (Figure S21; Table S1). At higher magnification, hexagonal pore channels can be seen face‐on, with a ∼6.0 nm diameter, consistent with the expected pore structure for **Joa‐COF‐1** (Figure 3d,e). The hexagonal channels are also observed side‐on in different projections (Figure 3f,g), indicating a preferential AA stacking. In some cases, the channels appear to be filled (Figure 3d), which may support the presence of quasi‐AB stacking modes. Tilting the COF particles in HR‐TEM allows to link the principal projections to each other (Figure 3h). The smaller lattice spacings observed in TEM images can be assigned to pore channels orientated off their principal projections with respect to the electron beam. A unique feature of **Joa‐COF‐1** is the tendency of the channels to separate into nearly free‐standing tubular nanostructures with a similar morphology to carbon nanotubes. When a suspension of the COF in hexane is treated by ultrasound followed by drop‐casting a suspension onto a TEM grid, some particles of COF disassemble into bundles consisting of 4 to 5 pore tubular nanostructures retaining exclusively a pore diameter of ∼6.0 nm (Figures 3i,k, S22 and S23) consistent with an AA stacking configuration. Since hexane is an inert solvent for boronate ester COFs, disassembly must occur purely through mechanical means, similar to carbon nanotubes and graphene exfoliation.^[^ ^37^ ^]^ This suggests that the crystallites of **Joa‐COF‐1** are loosely held together and can be readily separated by ultrasound into their constituent 1D structures along the direction of the hexagonal channels. A plausible explanation for this phenomenon could be the wavy structure of **Joa‐COF‐1**, which may favor stacking through layer complementarity at the expense of lateral growth, ultimately leading to the formation of these tubular nanostructures.

**Figure 3:**
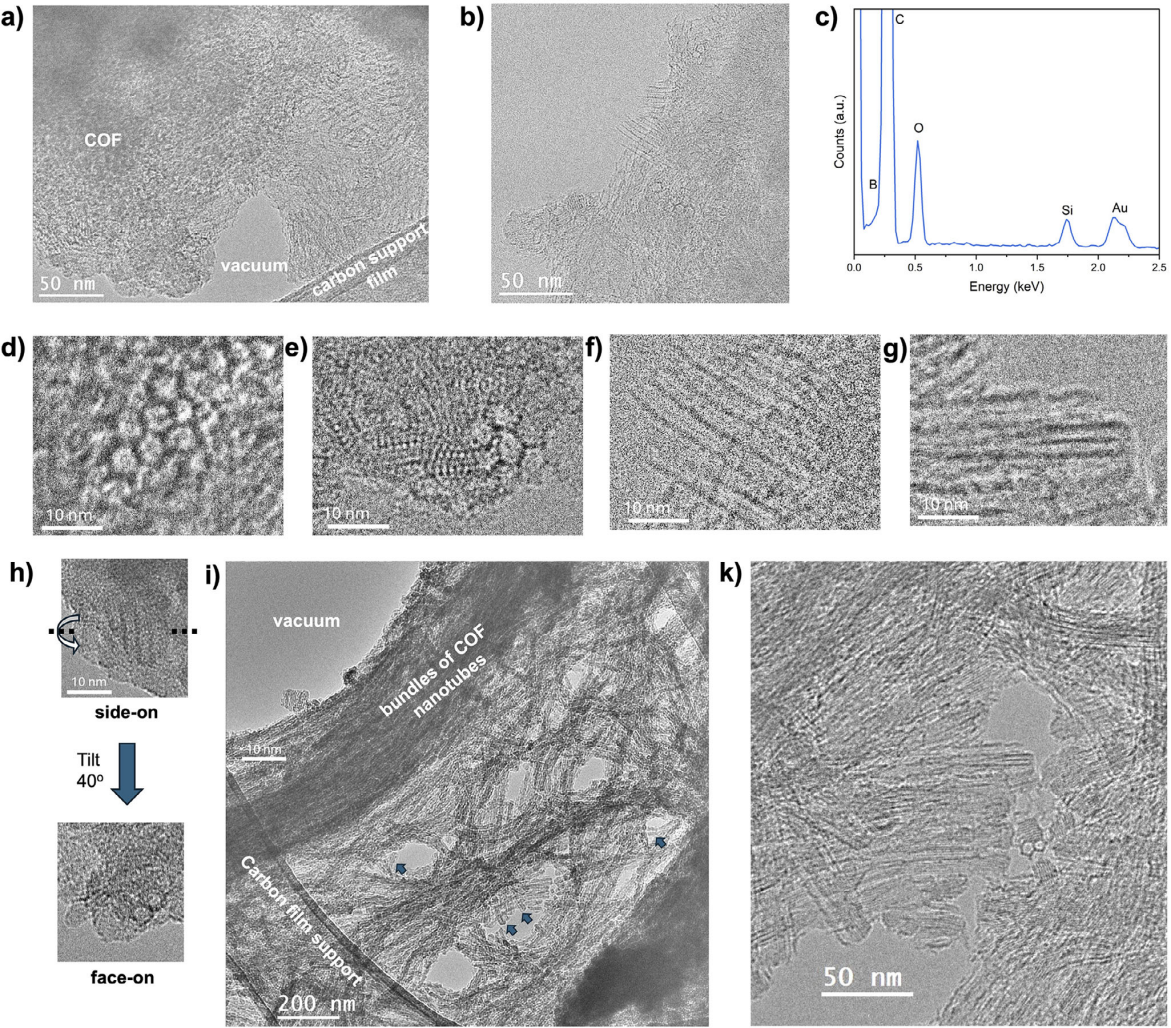
200 kV TEM analysis of **Joa‐COF‐1** a) and b) Large field of view HR‐TEM images of COF particles showing areas with different orientations of 6 nm channels (side‐on and face‐on orientations). c) The EDX spectrum of a COF particle shows C and O peaks (the B peak overlaps with the C peak and appears as a shoulder; the Si and Au peaks are from the TEM grid). d), e) face‐on and f), g) side‐on images of hexagonal channels in the COF structure. h) Tilting helps to reveal the relationship between the side‐on and face‐on projections of the same group of channels. i), k) Bundles of COF nanotubes are formed by disassembling **Joa‐COF‐1** particles by ultrasound (arrows in i) indicate sections of single COF nanotubes protruding from the bundles).

The PXRD pattern of **Joa‐COF‐1** exhibits several broad diffraction peaks (Figure 4a), consistent with the nanometric crystalline tubular domains observed by TEM (Figure 3), which disrupt long‐range ordering. Yet the peaks are consistent with the wavy honeycomb lattice of **Joa‐COF‐1**. Several potential crystal structures of **Joa‐COF‐1** were explored using density functional theory (DFT). Large pore COFs are challenging to address computationally, as, due to their flexibility, a wide variety of intra‐ and interlayer arrangements may be obtained.^[^ ^38^ ^]^ In contrast, the waviness of the monolayer limits the stacking arrangements, which is probably connected to the observed local crystallinity. Two different stackings were found to be stable with DFT, namely AA and quasi‐AB (Figures 4d,e; Table S2). The AA stacking mode is more stable thermodynamically than the quasi‐AB mode (Table S3). Both the simulated PXRD patterns of the AA and quasi‐AB stacking modes match most of the experimental PXRD pattern (Figures 4a and S24), revealing hexagonal 2D unit cells with peaks at 1.5°, 2.6°, and 4.0°, corresponding to the (100), (110), and (210) reflections, respectively. Computer modelling identifies the experimental peak found at 12.7° as the signature peak for quasi‐AB stacking, indicating that offset‐layer stackings are also quantitatively populated in the experimental samples.

**Figure 4:**
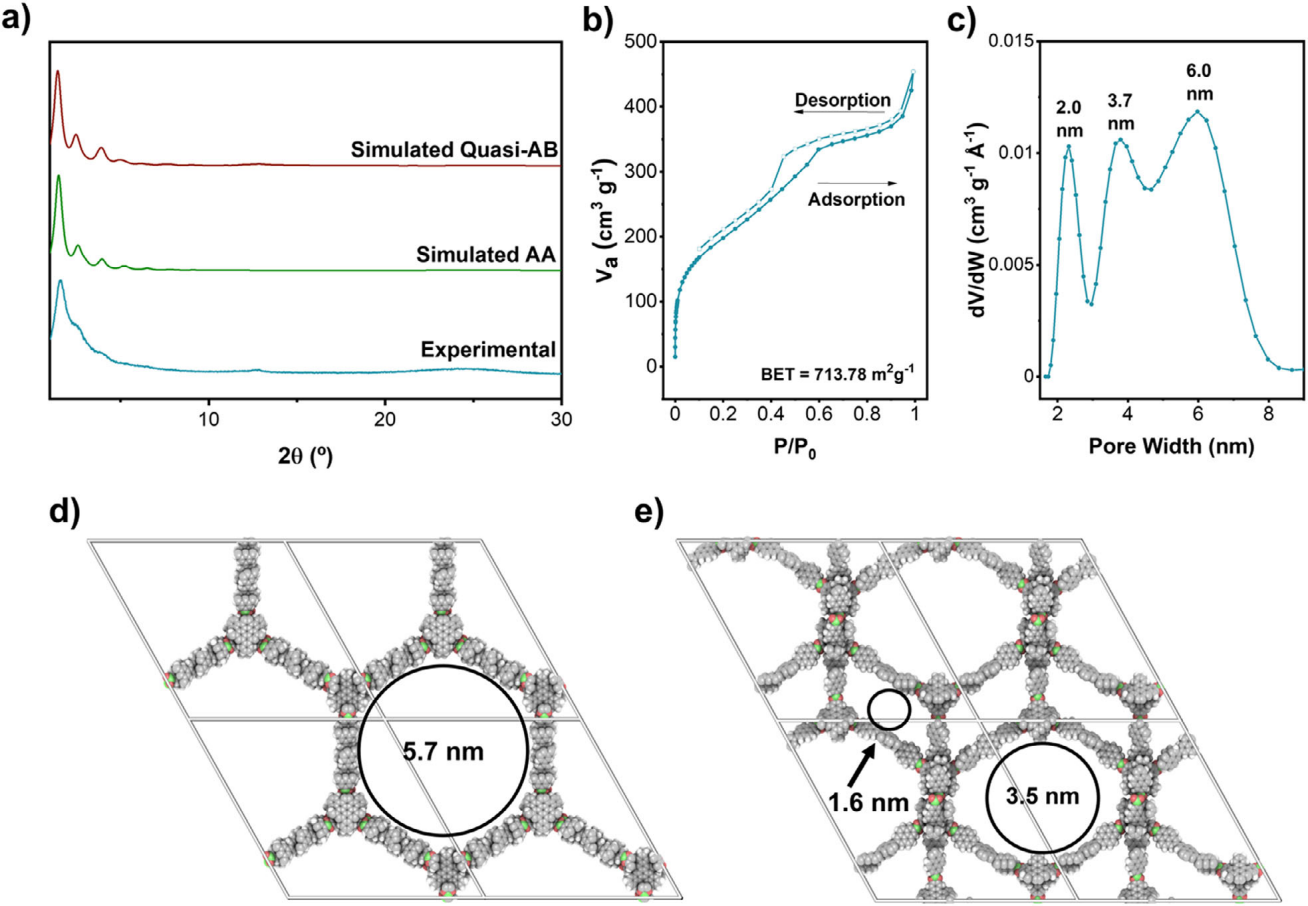
a) Experimental and simulated PXRD patterns for **Joa‐COF‐1**. b) Nitrogen adsorption and desorption isotherm at 77 K of **Joa‐COF‐1**. c) DFT pore size distribution of **Joa‐COF‐1**. Calculated d) AA and e) quasi‐AB packing modes and reference spore sizes of **Joa‐COF‐1**.

Surface area and pore size distribution of **Joa‐COF‐1** were established by nitrogen adsorption measurement at 77 K. **Joa‐COF‐1** exhibits a reversible type IV isotherm with microporous and mesoporous regions. The Brunauer–Emmet–Teller (BET) surface area is 714 m^2^g^−1^ (Figure 4b). The DFT model pore size distribution reveals three different pores of 2.0, 3.7, and 6.0 nm in **Joa‐COF‐1** samples (Figure 4c). The large experimental 6.0 nm pore agrees with the 5.7 nm calculated pores for the AA packing mode (Figures 4c,d, and S25; Table S4), whereas the 2.0 and 3.7 nm experimental pores agree with the calculated 1.6 and 3.5 nm pores for the quasi‐AB packing mode (Figures 4c,e, and S25; Table S4).

The presence of both AA and quasi‐AB packing modes in **Joa‐COF‐1** is supported by various measurements. TEM images reveal empty hexagonal channels consistent with AA stacking (Figure 2e,f,h) and filled hexagonal channels indicative of quasi‐AB stacking (Figure 2d). Additionally, the simulated PXRD patterns for the AA and quasi‐AB configurations of **Joa‐COF‐1** closely match the experimental pattern in both cases (Figures 4a and S24). The experimental pore size distribution further supports this duality, showing three pore sizes consistent with the combined contributions of the AA and quasi‐AB configurations. Yet, tubular motifs observed by TEM correspond to an AA configuration. This is consistent with the lower symmetry of quasi‐AB stacking that resembles AA in projection. As a result, the TEM contrast of the quasi‐AB domains will be difficult to distinguish in the polycrystalline particles of **Joa‐COF‐1**.

In summary, we have developed the synthesis of a diboronic acid linker with a 2.7 nm terpyrenyl backbone (**1**). The condensation between terpyrenyl linker **1** and the non‐planar HBC **2** gives rise to **Joa‐COF‐1**, a mesoporous wavy 2D COF with 6 nm pores. The PXRD pattern, the pore size distribution, and HR‐TEM images confirm that **Joa‐COF‐1** is constituted by small crystalline domains with local crystallinity, in which both AA and quasi‐AB packing domains may coexist. The mild sonication of **Joa‐COF‐1** leads to the disaggregation of such domains into freestanding 4 to 5 pores tubular nanostructures, which combine a 1D morphology with 6 nm mesopores. Overall, this work opens the door to the synthesis of other large‐pore 2D COFs and of tubular porous nanostructures that may be useful in the preparation of hybrid materials encapsulating biological or synthetic macromolecules and to the preparation of novel nanostructures constituted by COFs.

## Supporting Information

Supporting Figures and Tables. Full experimental procedures with structural spectroscopic characterisation. The authors have cited additional references within the Supporting Information.^[^ ^28^, ^39^, ^40^, ^41^, ^42^, ^43^, ^44^, ^45^, ^46^, ^47^, ^48^, ^49^, ^50^, ^51^, ^52^ ^]^

## Conflict of Interests

The authors declare no conflict of interest.

## Supporting information



Supporting Information

## Data Availability

The data that support the findings of this study are available in the supplementary material of this article.
