# Supplementary material for: Tubular Nanostructures from Large‐Pore 2D Covalent Organic Frameworks
Source: Angew Chem Int Ed Engl. 2025 Apr 4;64(23):e202505935. doi: 10.1002/anie.202505935 (PMC12124442; doi:10.1002/anie.202505935)
Supplement: Supplementary file 1 — Supporting Information [file ANIE-64-e202505935-s001.pdf]

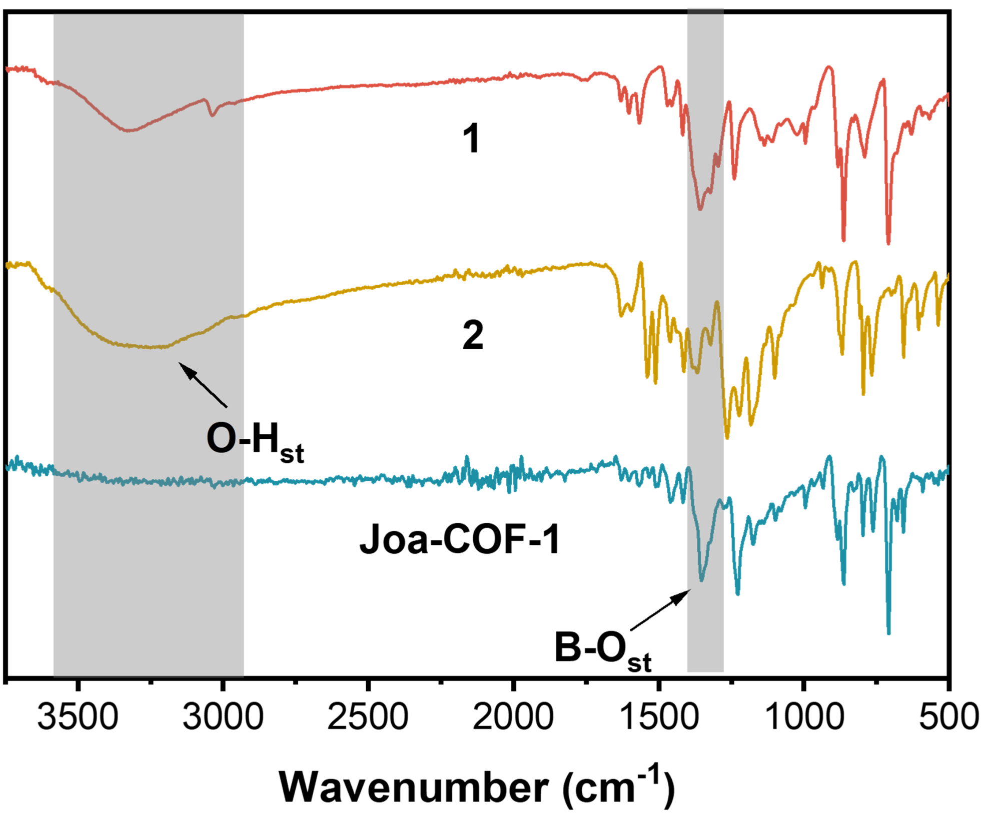

Figure S1. FTIR spectra of **1**, **2** and **Joa-COF-1**.

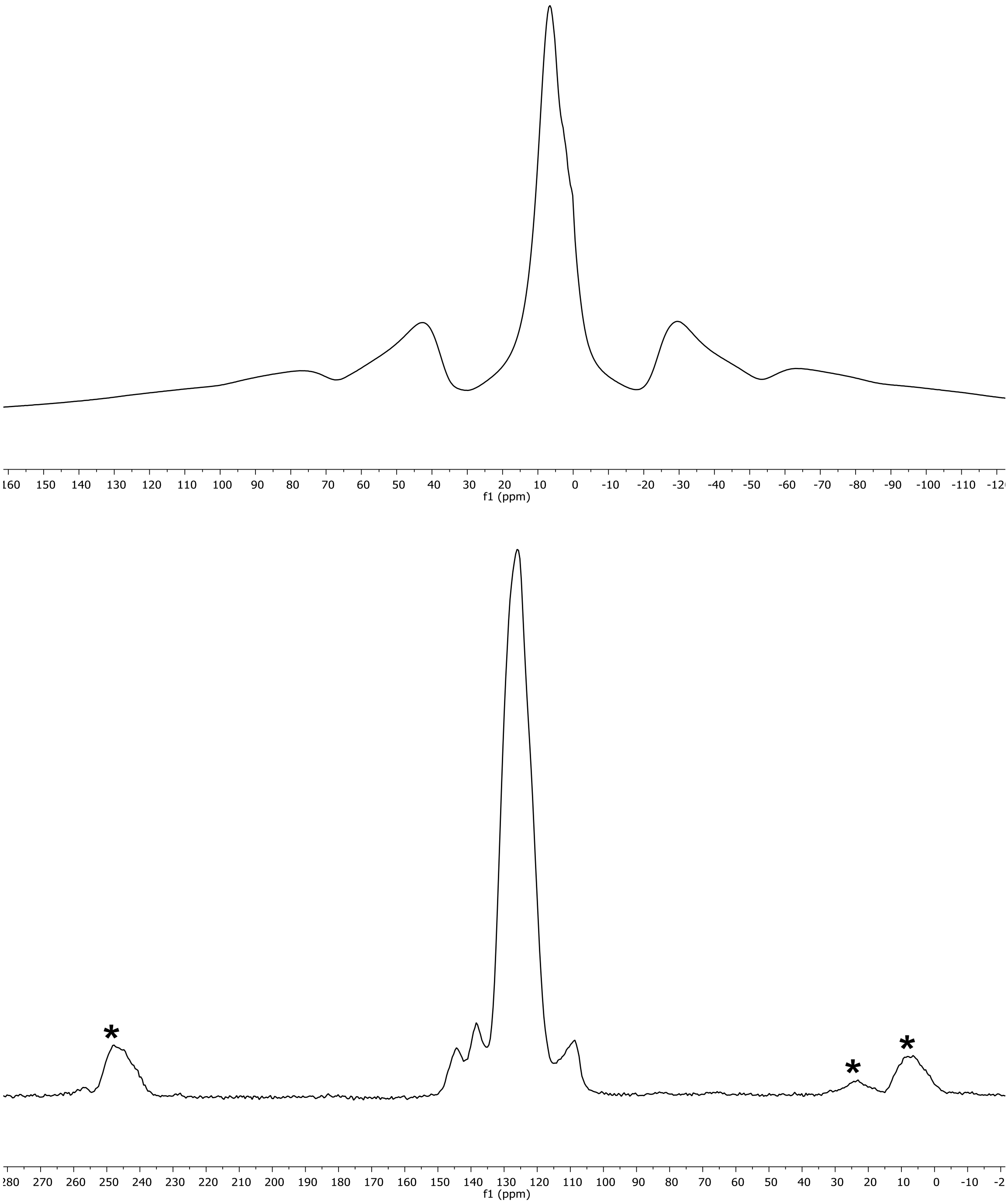

Figure S2. CP/MAS SS ^{1}H (top) and ^{13}C (bottom) NMR spectra of **Joa-COF-1** (* indicate spinning side bands).

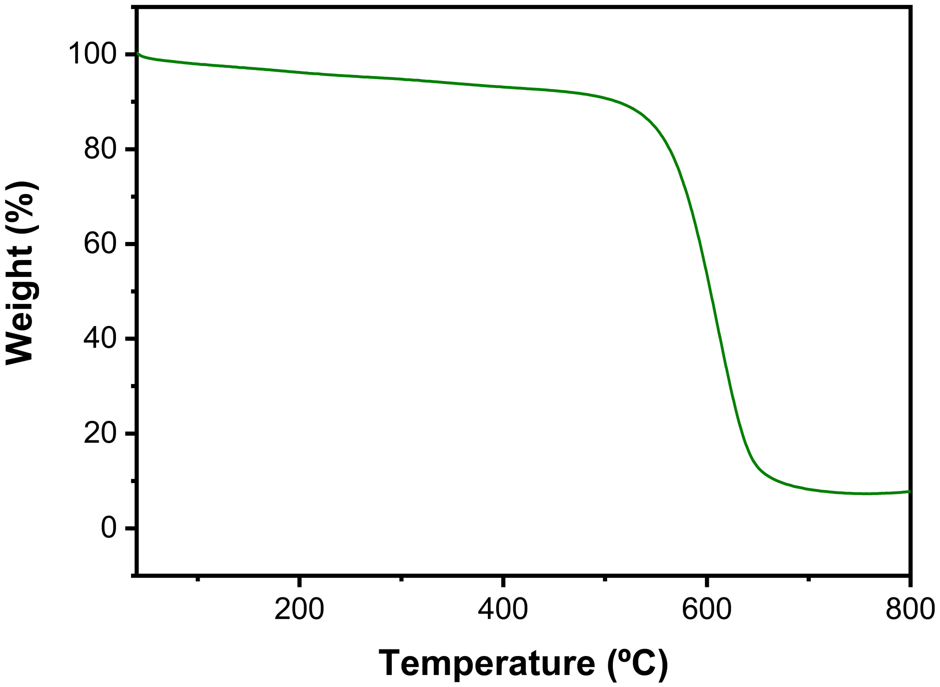

Figure S3. Thermogravimetric analysis of **Joa-COF-1** performed at 10 °C min^{-1} under N_2 (air was flushed after 800 ºC)

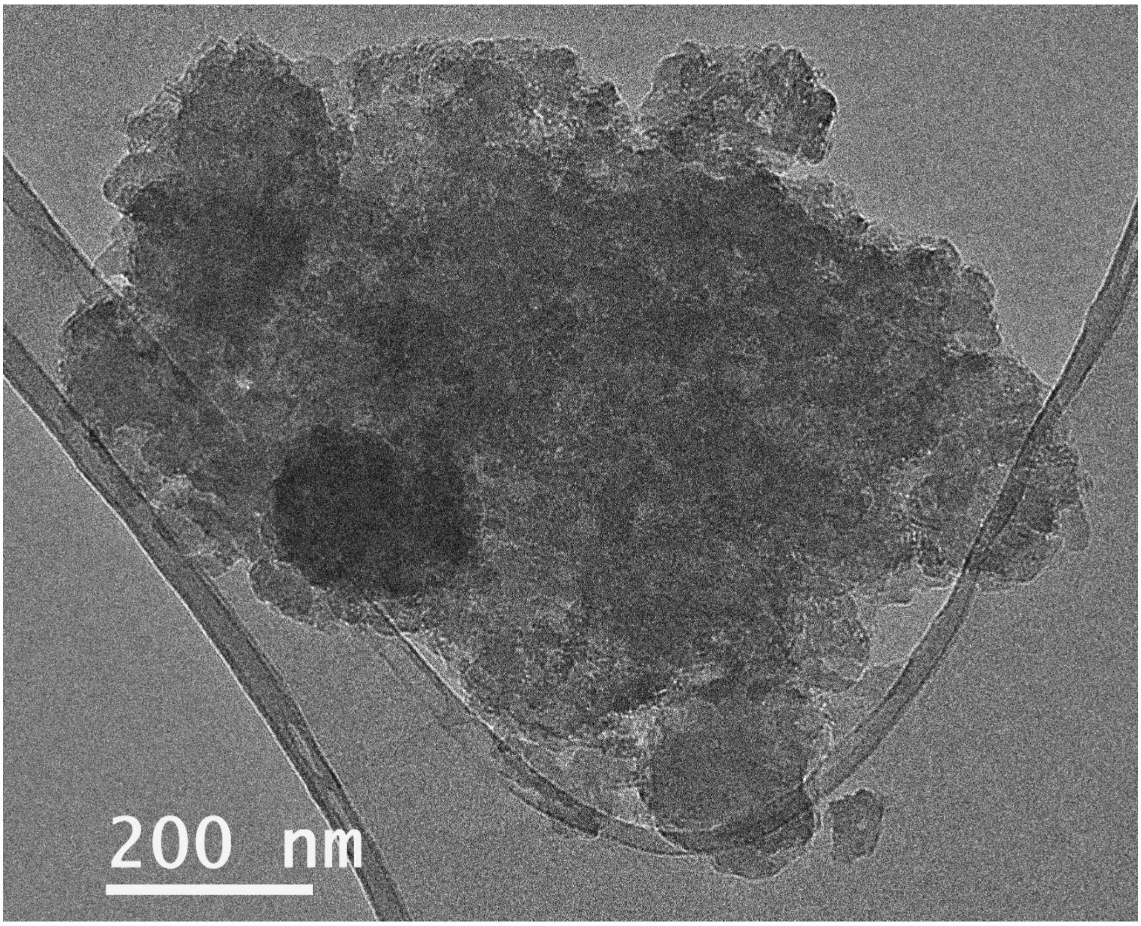

Figure S4. TEM micrograph of **Joa-COF-1**.

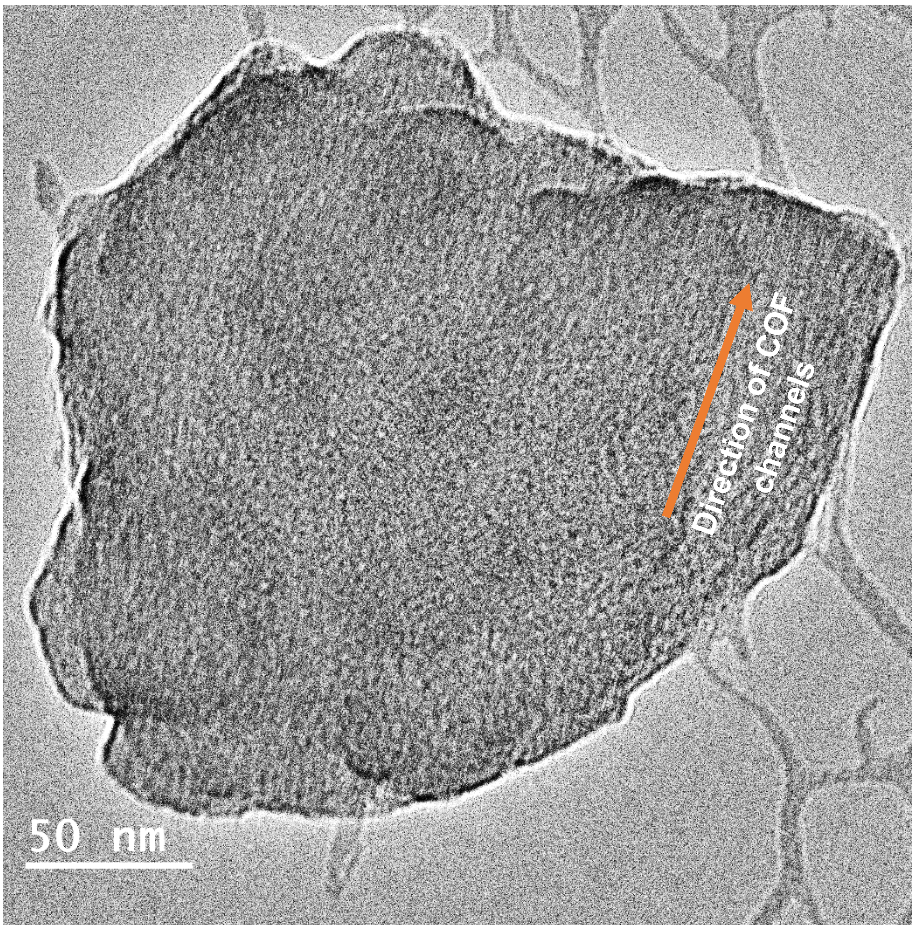

Figure S5. TEM micrograph of **Joa-COF-1**. The material is composed of a nanocrystalline powder consisting of particles with local crystalline order.

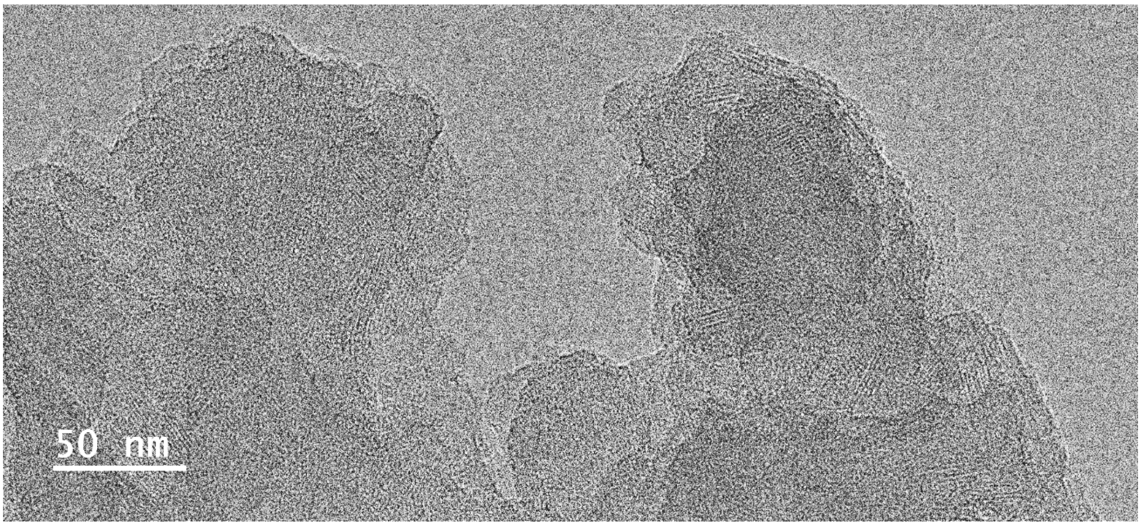

Figure S6. TEM micrograph of **Joa-COF-1**. Nanocrystalline domains of ~50 nm can be recognised by parallel lines in the microparticle.

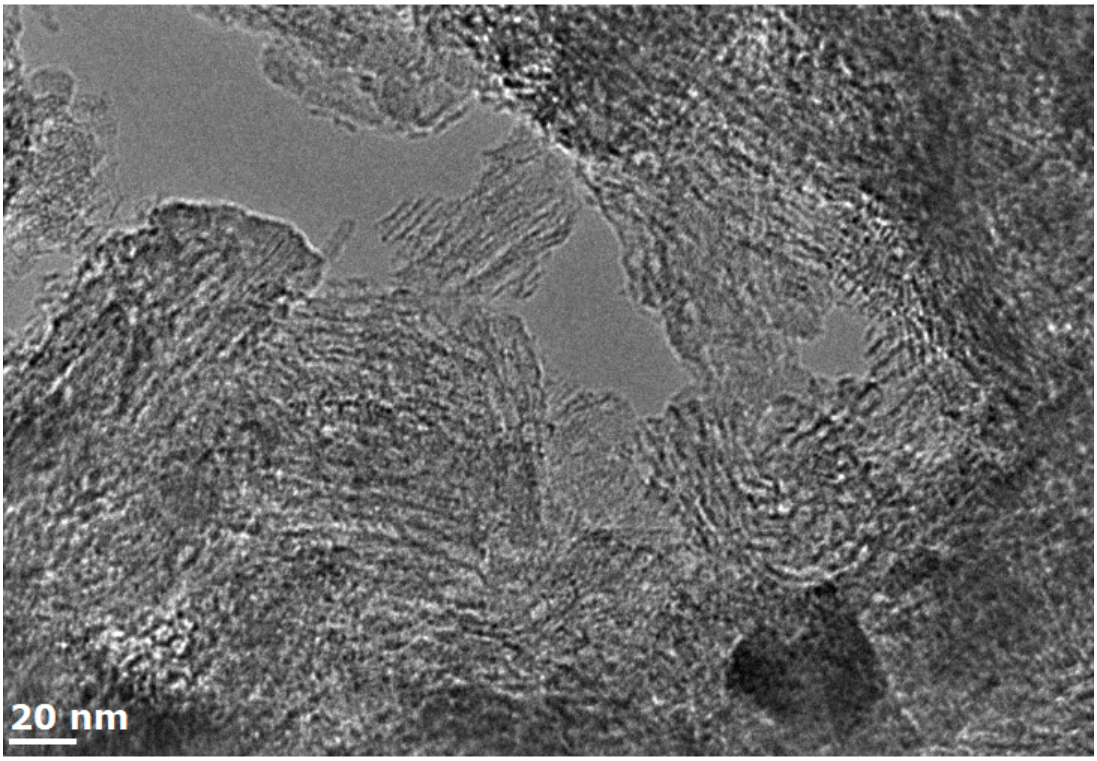

Figure S7. HR-TEM micrograph of **Joa-COF-1**.

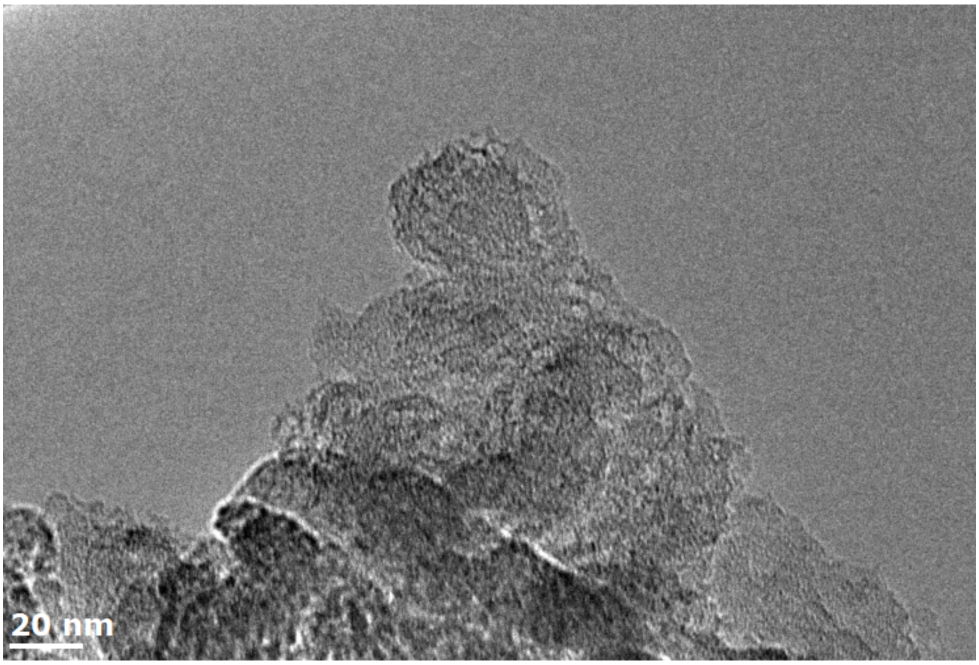

Figure S8. HR-TEM micrograph of **Joa-COF-1**.

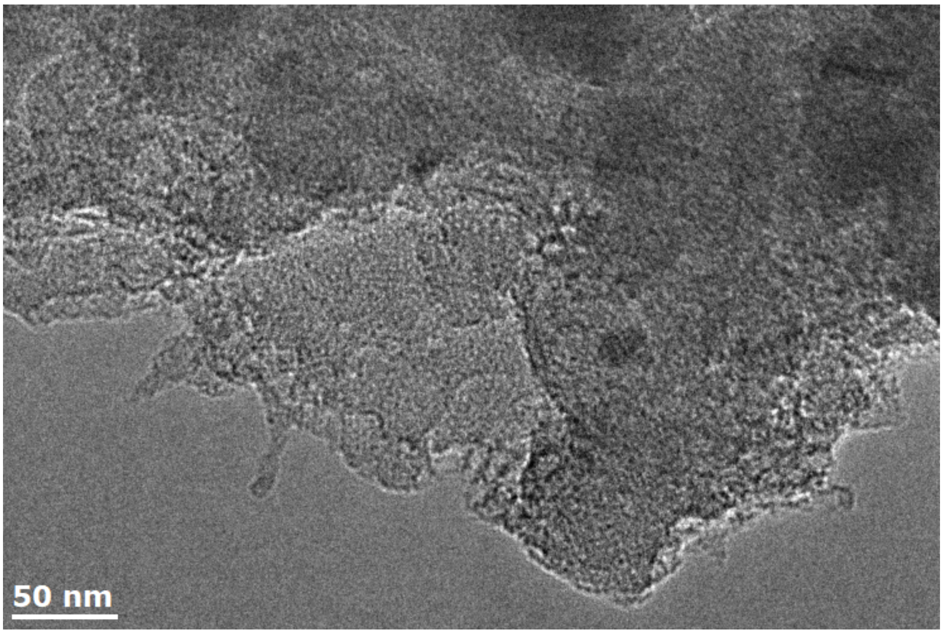

Figure S9. HR-TEM micrograph of **Joa-COF-1**.

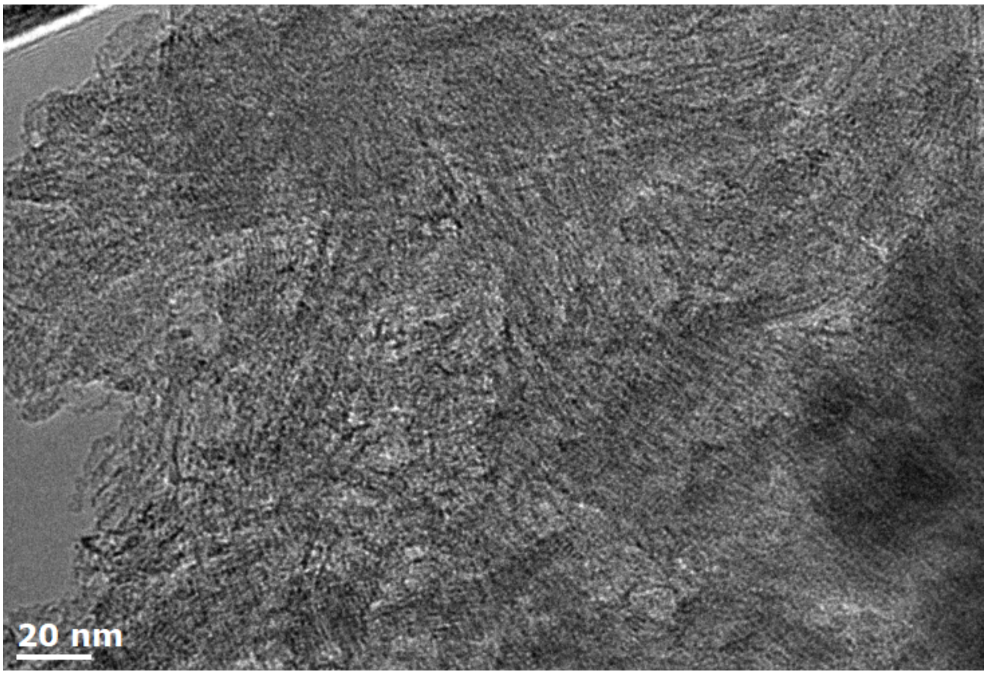

Figure S10. HR-TEM micrograph of **Joa-COF-1**.

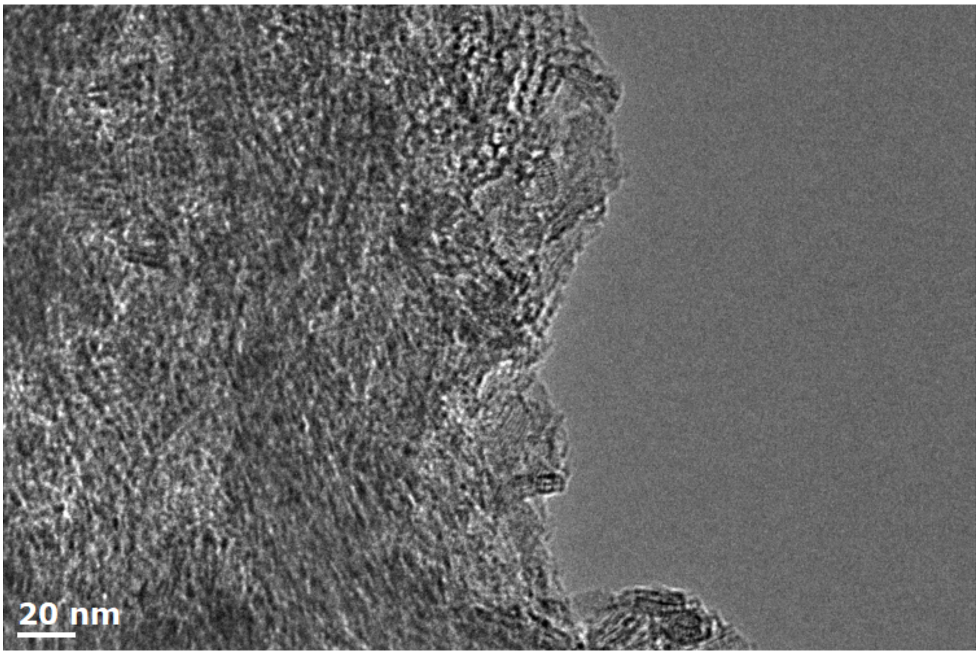

Figure S11. HR-TEM micrograph of **Joa-COF-1**.

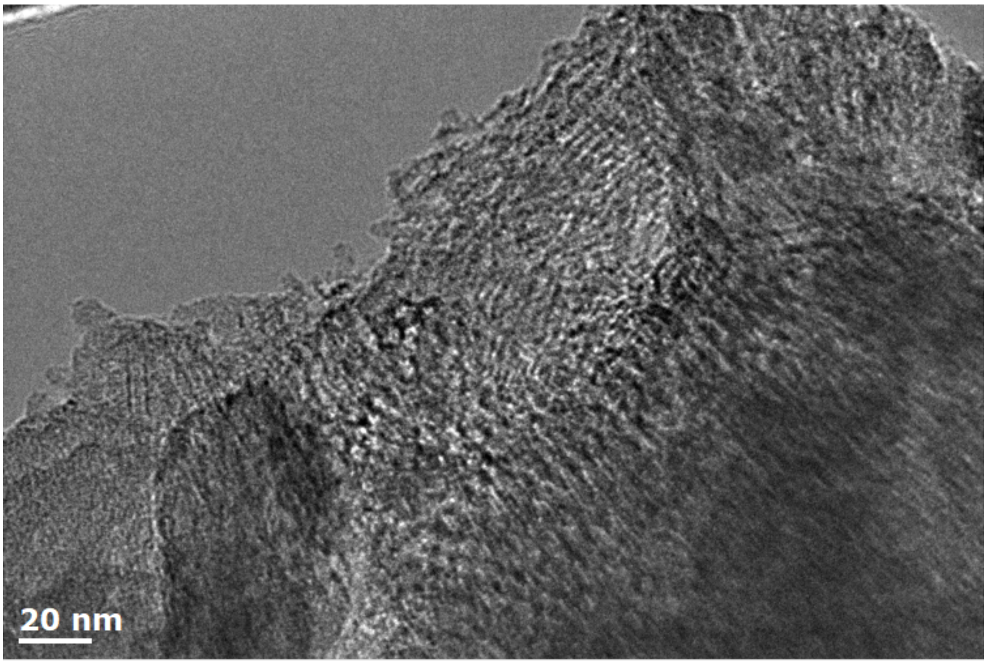

Figure S12. HR-TEM micrograph of **Joa-COF-1**.

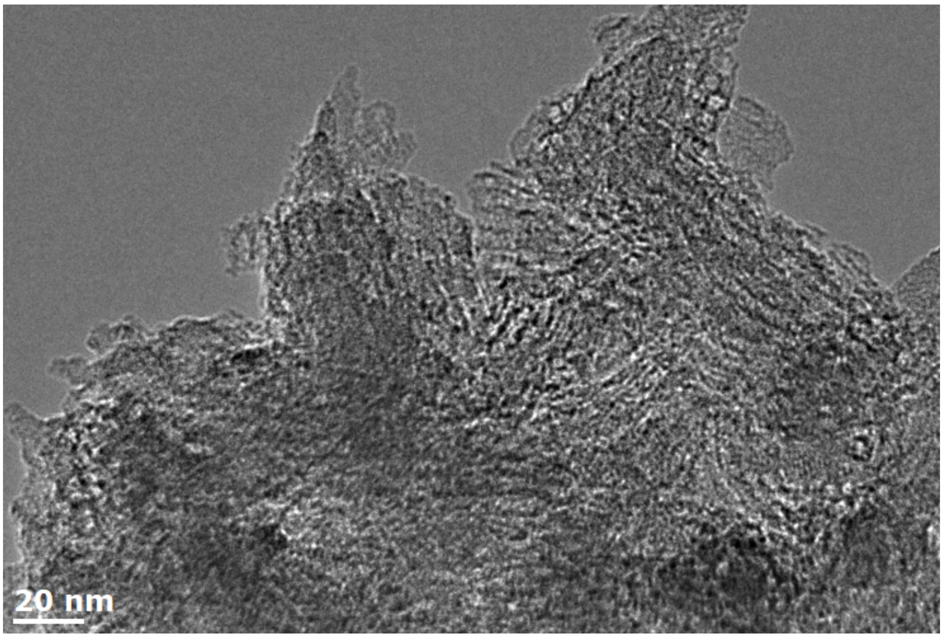

Figure S13. HR-TEM micrograph of **Joa-COF-1**.

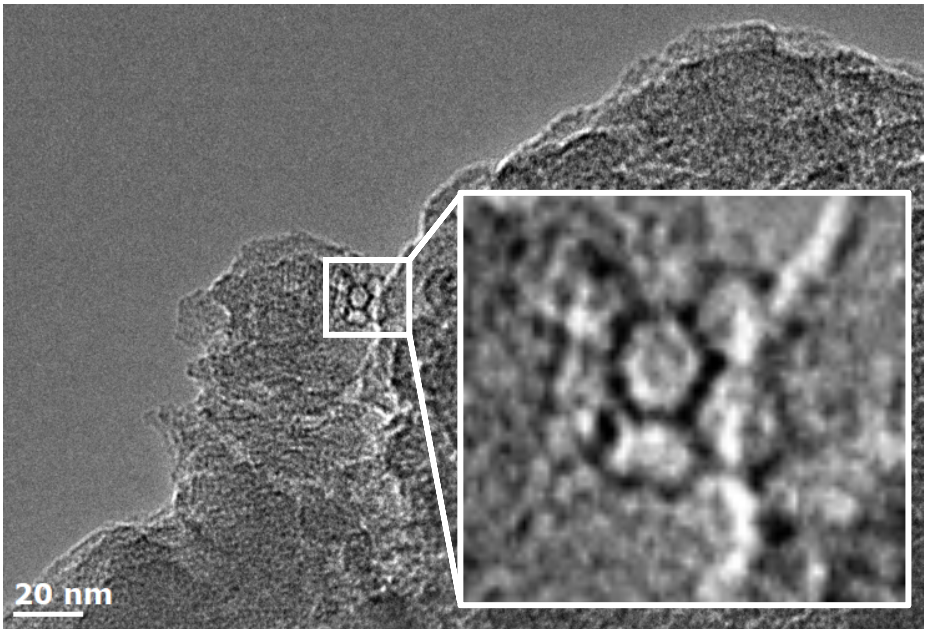

Figure S14. HR-TEM micrograph of **Joa-COF-1**. At higher magnification, hexagonal channels can be seen face-on.

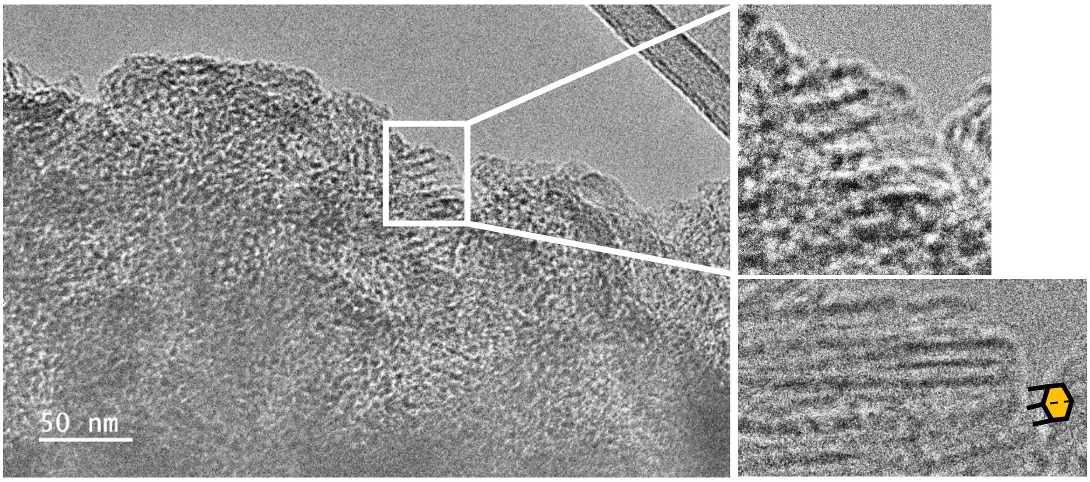

Figure S15. HR-TEM micrograph of **Joa-COF-1**. At higher magnification, hexagonal channels can be seen side-on. Side projection of the channel gives a dark line in the middle.

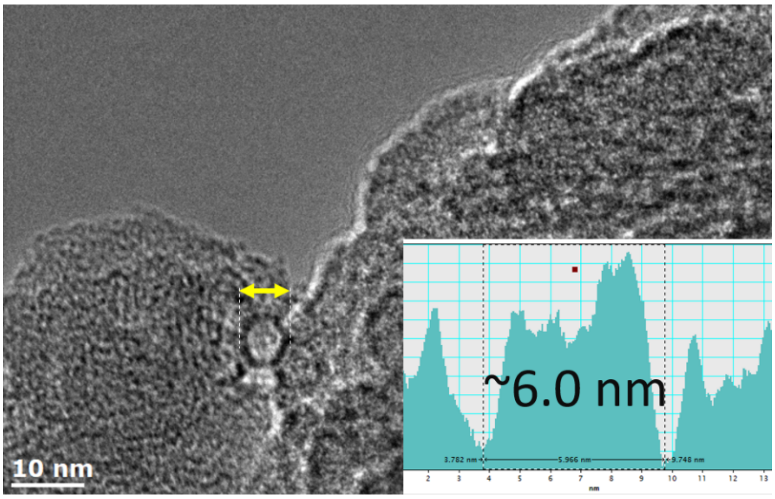

Figure S16. HR-TEM micrograph of **Joa-COF-1**. At higher magnification, hexagonal channels can be seen face-on. The channel exhibits an estimated pore size of 6.0 nm.

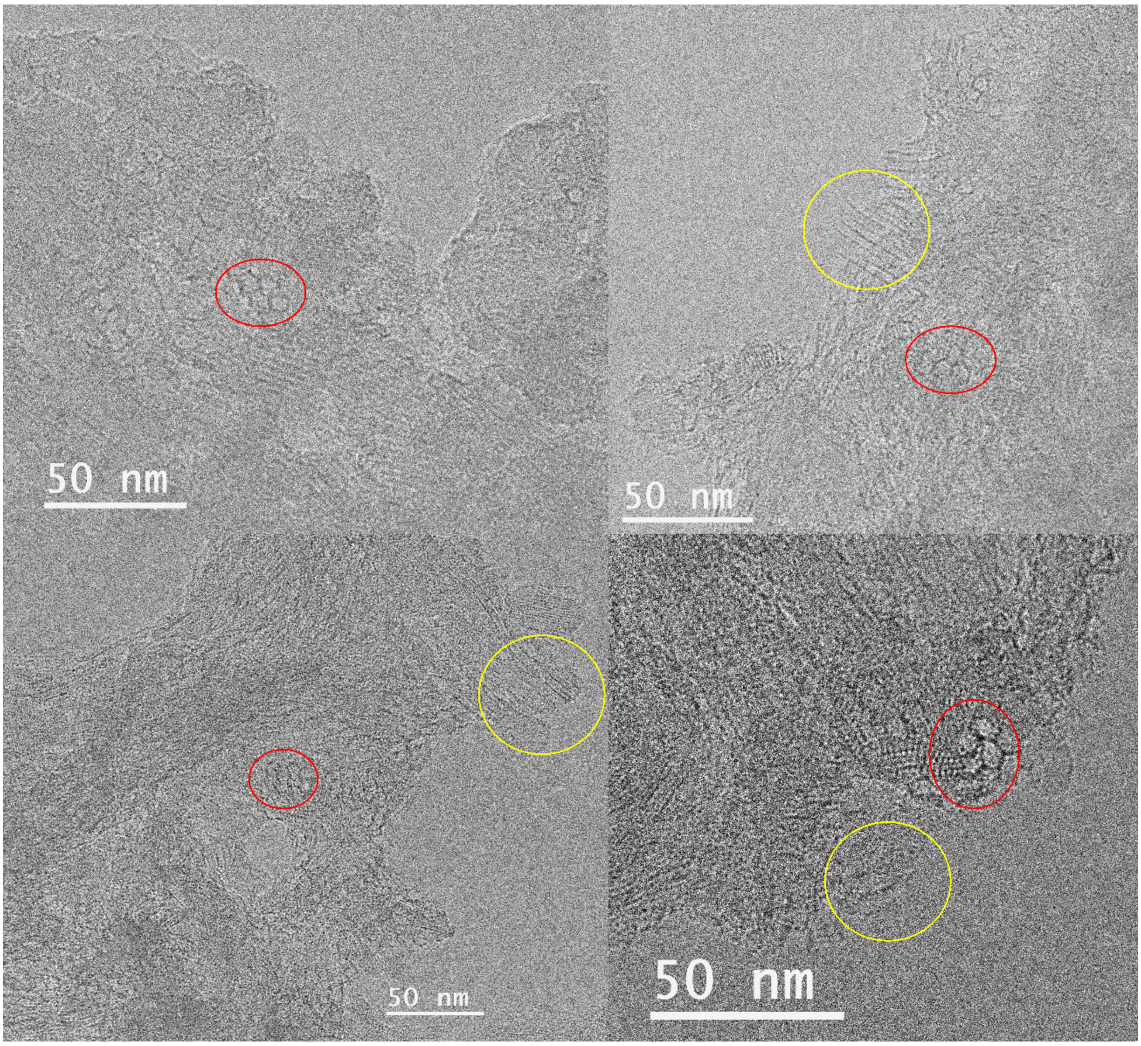

Figure S17. HR-TEM micrograph of **Joa-COF-1**. The areas marked with red circles are face-on hexagonal channels with 6.5 nm of size side-to-side approximately. The areas marked with yellow circles are side-on channels.

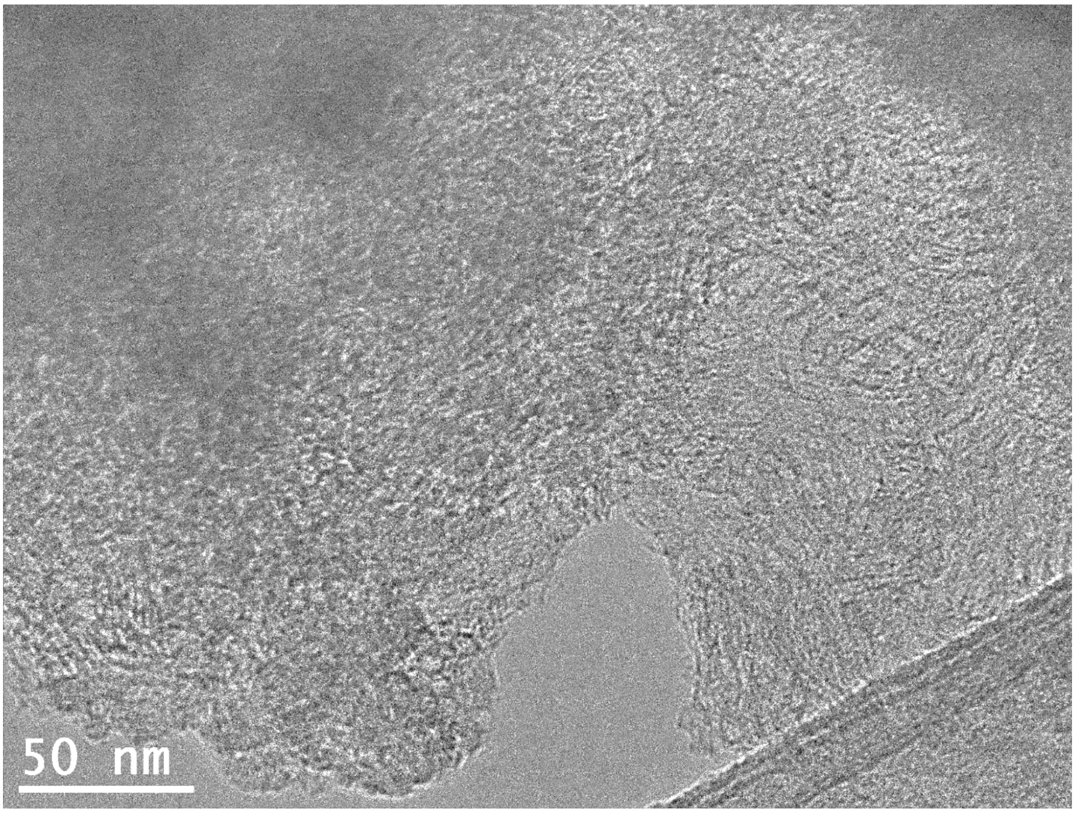

Figure S18. HR-TEM micrograph of **Joa-COF-1**.

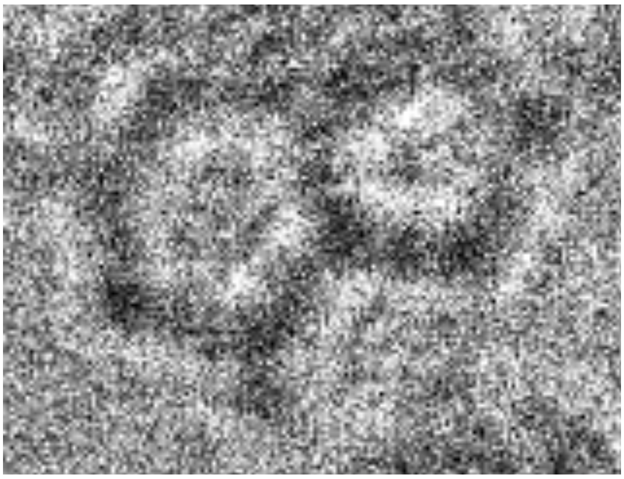

Figure S19. HR-TEM micrograph of **Joa-COF-1**. Channels appear to be filled.

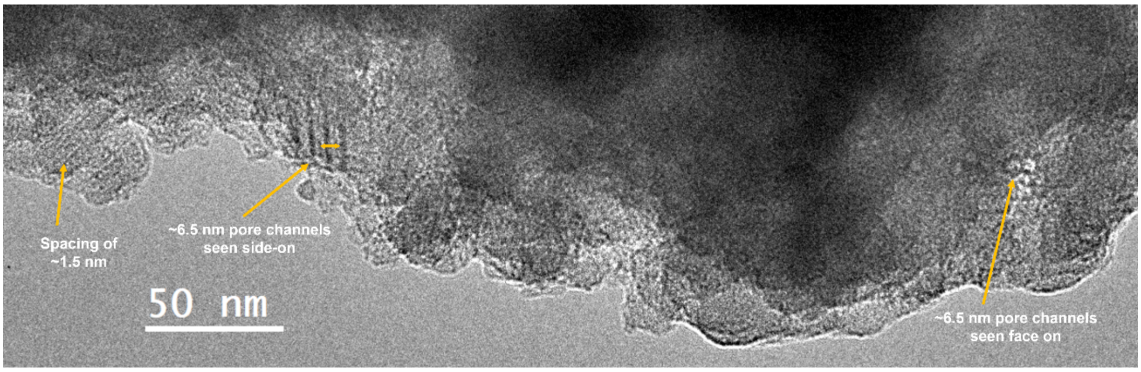

Figure S20. HR-TEM micrograph of **Joa-COF-1**. Smaller spacings observed in TEM images are likely pore channels orientated differently from the principal projection to the direction of the electron beam.

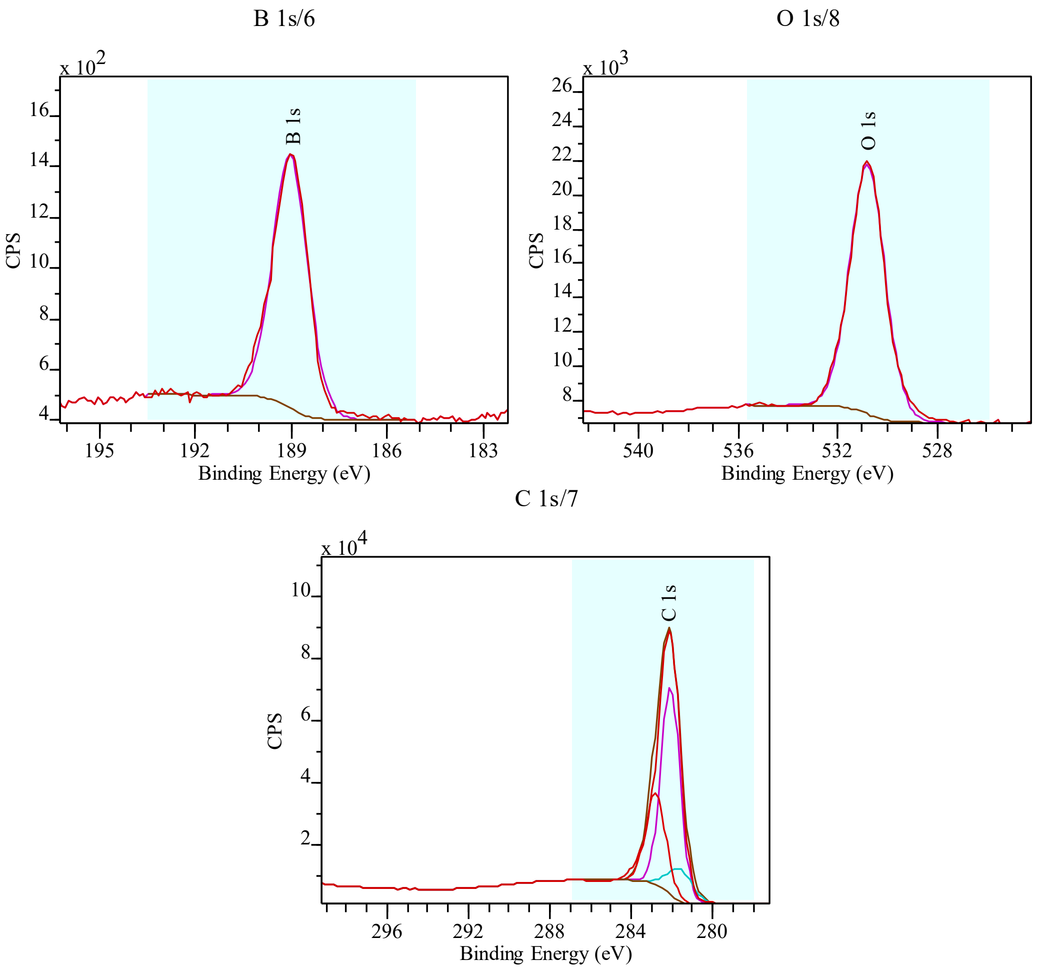

C-O 282.79 eV / C-C 282.07 eV / C-B 281.59 eV

Figure S21. XPS of **Joa-COF-1**.

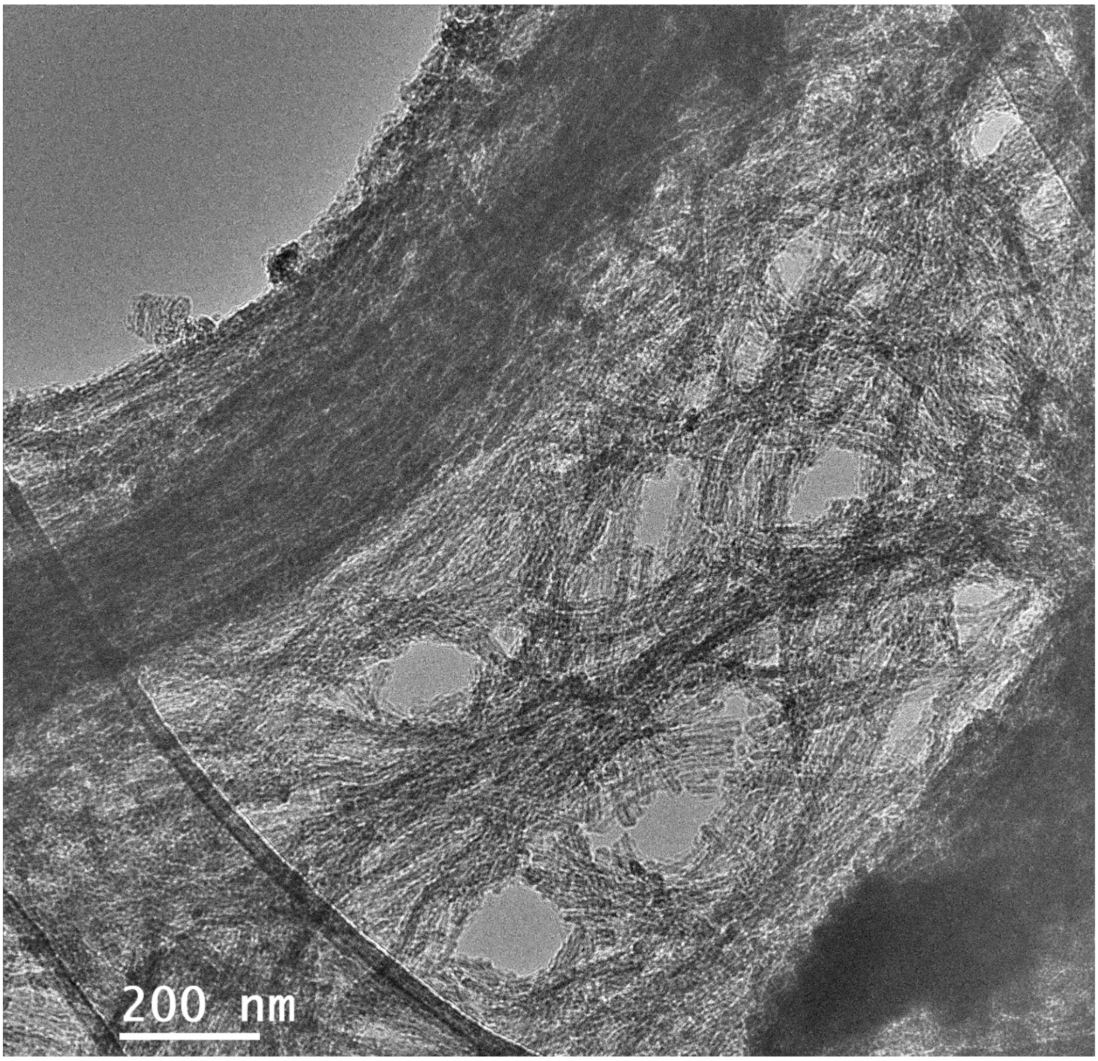

Figure S22. A complete HR-TEM micrograph of **Joa-COF-1** is shown in **Figure 2**. COF sample was prepared via sonication in hexane, and some areas were observed with separated pore channels, some in bundles, and some freestanding, similar to carbon nanotubes. Samples prepared via powder deposition did not show this feature. Some pore channels were imaged face-on (diameter ~6.5 nm), pore channels were imaged side-on (diameter ~6.2 nm), and some smaller d-spacing was observed (d=1.1 nm).

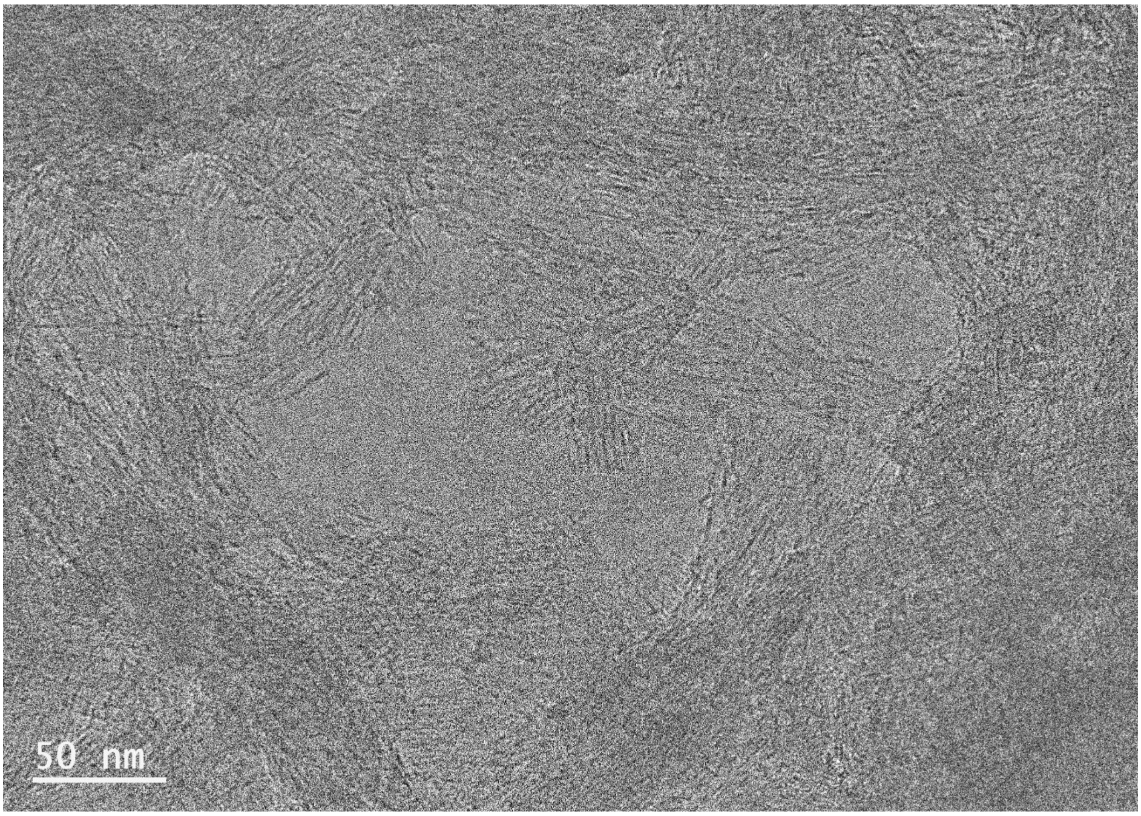

Figure S23. HR-TEM micrograph of **Joa-COF-1**. Exfoliated channels appear as spaghetti-like structures.

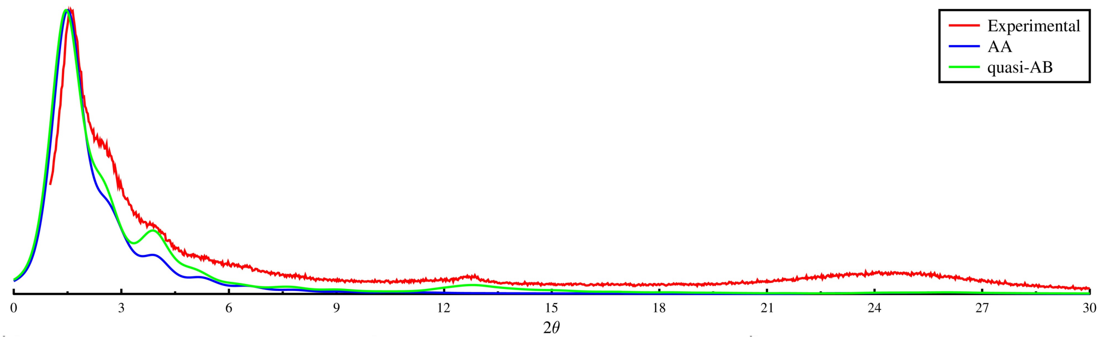

Figure S24. Normalized experimental and, simulated AA and quasi-AB PXRD patterns of **Joa-COF-1**.

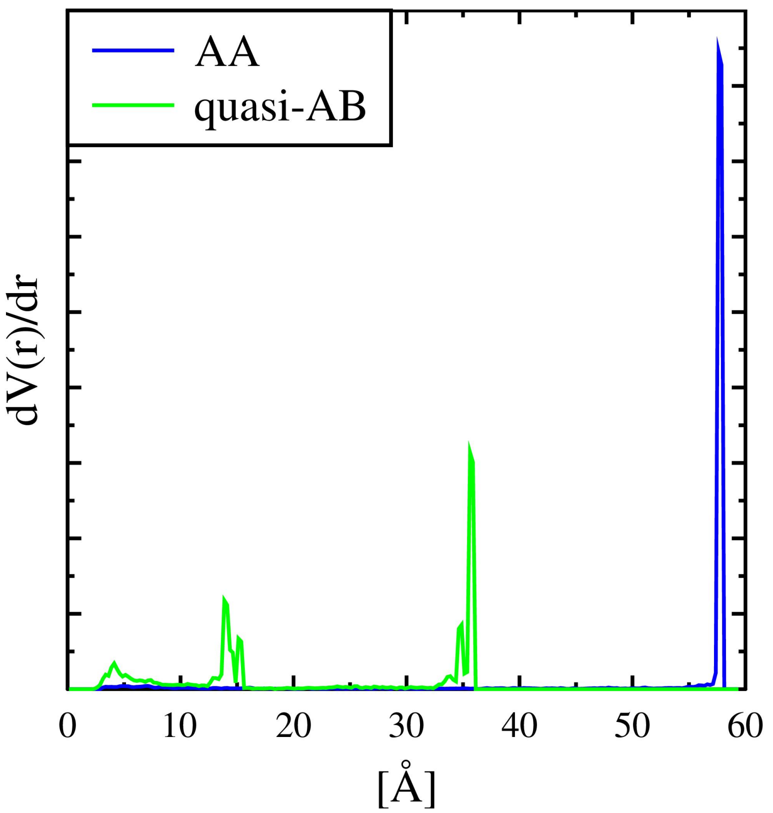

Figure S25. Pore size distribution of the AA and quasi-AB modelled stackings.

Table S1. Elemental composition of **Joa-COF-1** from elemental analysis (EA) and X-ray photoelectron spectroscopy (XPS). Deviations comparing experimental and theoretical values can be attributed to the presence of unreacted functional groups.

Technique	C	H	O	B	Total
Calculated (% wt)	88.74	3.35	5.91	2.00	100.00
EA (% wt)	89.35	3.56	-	-	92.91
XPS (% at)	91.66	-	6.76	1.59	100.00
XPS (% wt)	89.78	-	8.82	1.40	100.00

Table S2. Unit cell parameters of **Joa-COF-1** DFT models for a monolayer and bulk systems with two different stackings.

	a (Å)	b (Å)	c (Å)	α (°)	β (°)	γ (°)
1 Layer	69.42	69.79	493.23	90	90	120.01
AA	68.99	68.24	8.01	86.26	94.51	121.07
quasi-AB	72.23	68.19	7.55	87.14	93.62	121.1

Table S3. Binding energies (BE) from DFT calculations. PBE+MBD/light Hamiltonian.

	Total E (eV)	BE / layer (kcal mol^{-1})	BE / atom (meV)
Monolayer	-279065.251	NA	NA
AA	-558149.179	-215.346	-25.51
quasi-AB	-558137.599	-81.832	-9.7

Table S4. Computed porosity data for **Joa-COF-1**. The porosity, surface area and internal volume of predicted structures was calculated using PoreBlazer v4.0.

Stacking	Density (g/cm^3)	Pore Limiting Diameter (Å)	Surface Area (m^2/g)	Pore Accessible Volume (cm^3/g)
AA	0.335	57.3	1745	2.34
quasi-AB	0.339	35.5	3290	2.31

Experimental Procedures

Reagents for synthesis were, if not otherwise specified, purchased from Aldrich, TCI or Fluorochem. Column chromatography was carried out using Silica gel 60 (40-60 µm) from Scharlab. Analytical thin layer chromatography (TLC) was done using aluminum sheets (20x20 cm) pre-coated with silica gel RP-18W 60 F254 from Merck. UV-active compounds were detected with a UV-lamp from CAMAG at wavelength λ = 254 or 366 nm. 2,3,10,11,18,19-hexamethoxy-cata-hexabenzocoronene (HBC), 2,7-dibromopyrene and pyrene-2,7-bis(4,4,5,5-tetramethyl-[1,3,2]dioxaborolane) were synthesized according to reported methods.[1-2]

Joa-COF-1 was synthesized in a pre-scored 5 mL ampoule from Aldrich. For its synthesis and purification anhydrous 1,4-dioxane (99.8%), anhydrous acetone (99.8%), anhydrous hexane (97%) were purchased from Acros Organics. THF was dried using an Innovative Pure Solve solvent purification system and mesitylene (97%) was obtained from Acros Organics and dried with molecular sieves.
NMR spectra in solution were recorded on Bruker Avance 400 spectrometer at room temperature K using partially deuterated solvents as internal standards. Coupling constants (J) are denoted in Hz and chemical shifts (δ) in ppm. Multiplicities are denoted as follows: s = singlet, d = doublet, t = triplet, m = multiplet, br = broad.

Solid-State CP/MAS NMR spectra were recorded on a Bruker Avance III 400 MHz NMR spectrometer at a MAS rate of 12 kHz and a CP contact time of 2 ms.
Matrix Assisted Laser Desorption Ionization (coupled to a Time-Of-Flight analyzer) experiments (MALDI-TOF) were recorded on Bruker REFLEX spectrometer in POLYMAT.

ATR-FTIR spectra were recorded on a Bruker ALPHA ATR-IR spectrometer. TA Instruments Discovery system was used to perform the thermogravimetric analysis (TGA) using a 10 °C min^{-1} heating rate under a nitrogen flow, which was changed to oxygen from 800 °C.

The powder X-ray diffraction (PXRD) patterns were collected by using a PHILIPS X'PERT PRO automatic diffractometer operating at 40 kV and 40 mA, in theta-theta configuration, secondary monochromator with Cu-Kα radiation (λ = 1.5418 Å) and a PIXcel solid state detector (active length in 2θ = 3.347°). Data were collected from 1 to 50° 2θ (step size = 0.026 and time per step = 300 s, total time 40 min) at room temperature. A variable divergence slit, giving a constant 4.0 mm area of sample illumination, was used.

The pore structure was evaluated by nitrogen sorption isotherms, measured at 77 K with a Micromeritics 3Flex apparatus. The samples were degassed in an Autosorb station at and 10^{-6} Torr at 100 °C prior to analysis. Surface area, pore size and volume values were calculated from nitrogen adsorption-desorption isotherms (77 K). Specific surface area (SA) was calculated by multi-point Brunauer-Emmett-Teller (BET) method. Total pore volume was taken at P/P0=0.96. Pore size distribution

was analysed by using the solid density functional theory (NLDFT) for the adsorption branch by assuming a cylindrical pore model.

Electron Microscopy. COF powder was deposited dry by pressing a lacey carbon-coated copper TEM grid (Agar Scientific) directly onto the COF powder and removing excess material mechanically shortly prior to HR-TEM imaging to avoid hydrolysis by atmospheric moisture. Alternatively, COF material was dispersed in n-hexane (Fischer Scientific, >95%, ~ 1 mL) using ultrasonic agitation (Agar Sonomatic, 100 W, HF frequency 30 kHz) over 5 minutes, and the resultant suspension was drop cast (5 drops) onto a TEM grid. This dispersion approach led to the disassembly of COF particles into COF nanotubes. High Resolution Transmission Electron Microscopy (HRTEM) analysis was performed on a JEOL2100F operating at 200 kV, equipped with a Gatan K3-IS electron detecting camera. Electron flux was minimised (200-1000 $e^{-}nm^{-2}s^{-1}$) to ensure a low electron fluence in order to mitigate electron beam induced sample damage. The tilt series was taken using a JEOL single-tilt holder, acquiring images at 5° tilt intervals. All TEM data was processed using Gatan Microscopy Suite version 3.5. EDX data was collected using an Oxford Instruments XMax 80 detector, and was processed using INCA X-ray microanalysis software.

Computer Modelling. The Tight Binding (TB) calculations were used to initially explore different stackings of the bulk COF. For this, the matsci parameter set with dispersion corrections from D4 was used within the DFTB+ program.[3-5] DFT calculations were performed with PBE[6-8] functional augmented with Many Body Dispersion corrections[9-10] using the software FHI-AIMS[11-13] with "light" numerical orbitals with the frozen core approximation with a -200 eV cutoff value. PoreBlazer v4.0[14-15] was used to compute the porosity of the simulated structures with a virtual nitrogen molecule and 10000 samples for surface area calculation with a cublet size of 0.2 Å.

7,7''-bis(4,4,5,5-tetramethyl-1,3,2-dioxaborolan-2-yl)-2,2':7',2''-terpyrene (5)

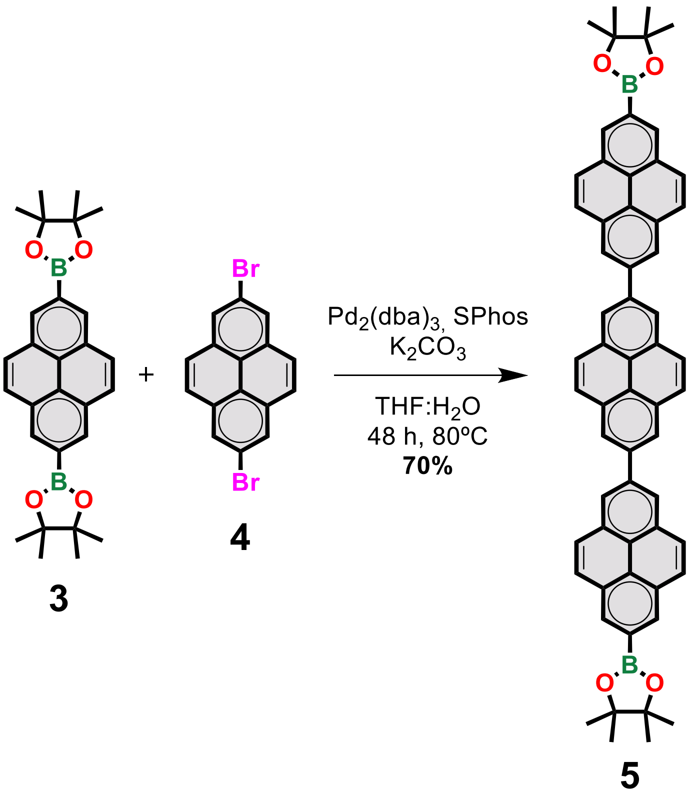

A solution of 2,7-dibromopyrene (**4**) (150 mg, 0.42 mmol), 4,4,5,5-tetramethyl-2-(pyren-2,7-yl)-1,3,2-dioxaborolane (**3**) (700 mg, 1.54 mmol) and potassium carbonate (125 mg, 0.90 mmol) in THF:water (5 mL: 1.2 mL) was purged with N_2 flow for 20 min. Tris(dibenzylideneacetone)dipalladium(0) ($Pd_2(dba)_3$) (38.4 mg, 0.042 mmol) and SPhos (34.5 mg, 0.084 mmol) were added and the mixture was heated at 80 °C overnight. Water was added and the precipitate was collected. It was washed with water (100 mL), methanol (100 mL), and diethyl ether (100 mL). The grey product was dissolved in chloroform and passed through a pad of celite. The organic phase was concentrated, and methanol was added producing a pale yellow solid. It was collected obtaining the desired product as a pale-yellow powder (251 mg, 70%).

^{1}H NMR (400 MHz, chloroform-*d*) δ 8.73 (s, 4H), 8.71 (s, 4H), 8.69 (s, 4H), 8.30 (s, 4H), 8.23 (d, *J* = 9.0 Hz, 4H), 8.19 (d, *J* = 9.0 Hz, 4H), 1.49 (s, 24H).

^{13}C NMR (101 MHz, chloroform- *d*) δ 142.98, 139.68, 139.37, 132.30, 131.83, 131.62, 130.48, 128.35, 128.17, 127.49, 126.34, 126.19, 124.86, 124.46, 123.95, 84.21, 25.04.

MS (MALDI-TOF) (m/z): calculated for $C_{60}H_{48}B_2O_4$: 854.374; found: 854.363 $[M]^+$

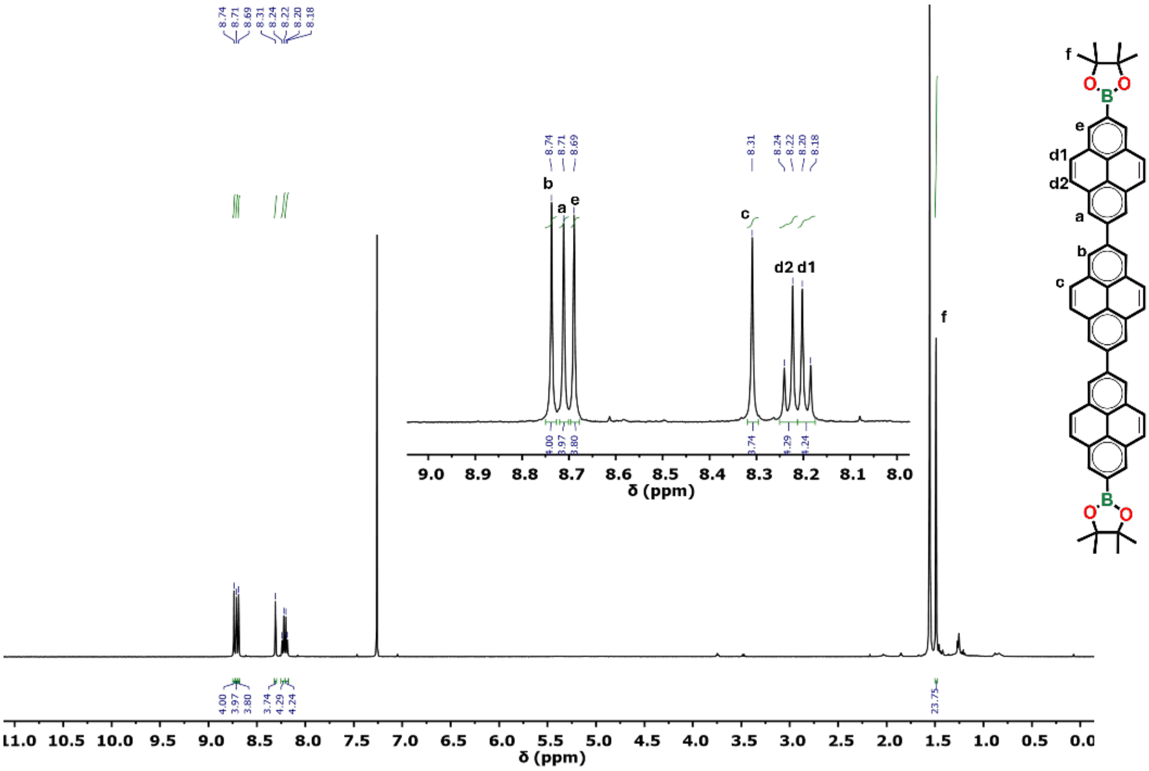

Figure S26. ^{1}H-NMR spectrum of compound **5**.

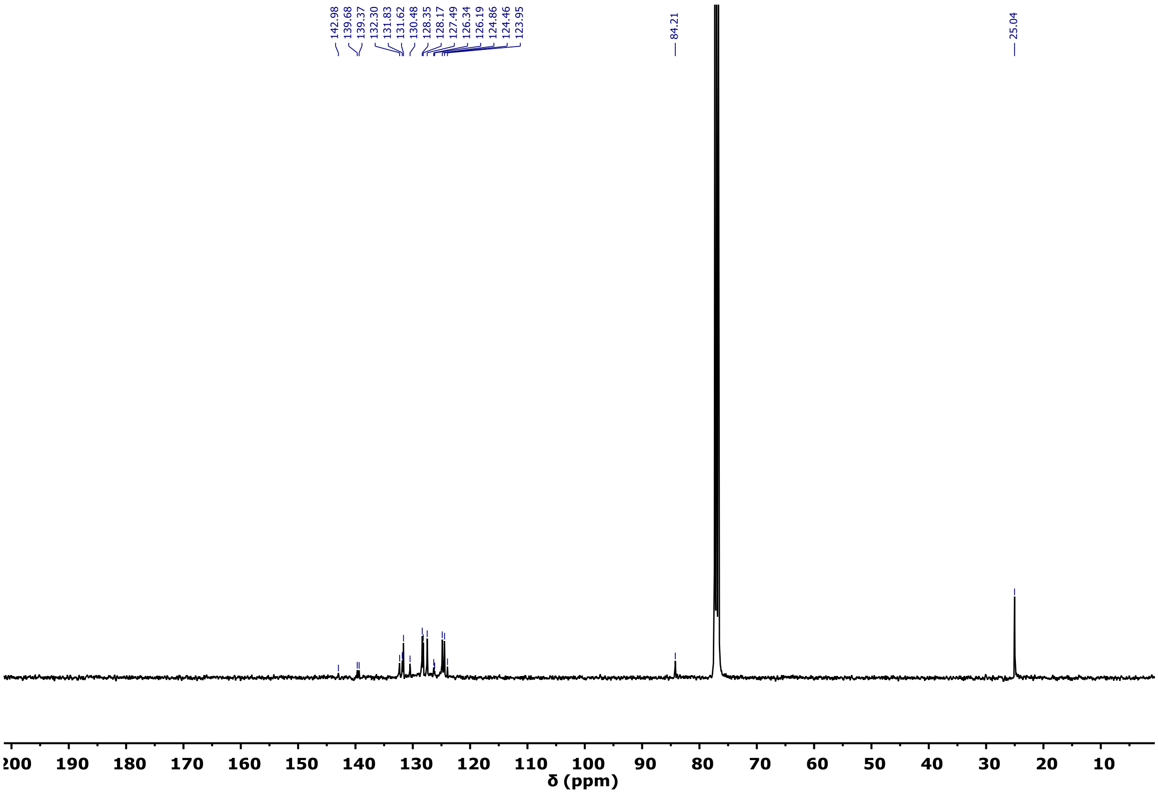

Figure S27. ^{13}C NMR spectrum of compound **5**.

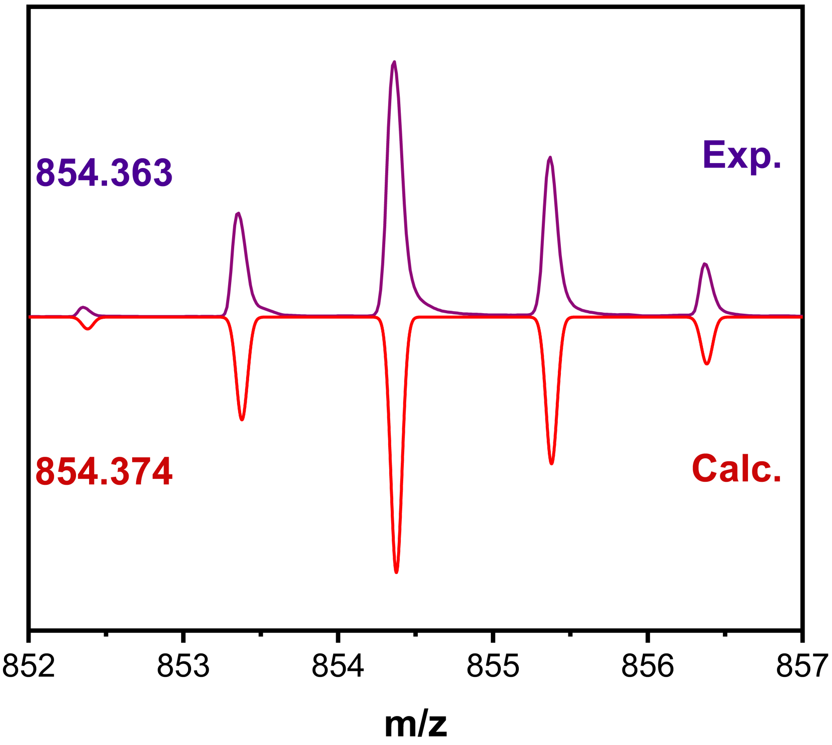

Figure S28. MS spectrum of compound **5**.

[2,2':7',2''-terpyrene]-7,7''-diyldiboronic acid (Terpy-B(OH)₂) (1)

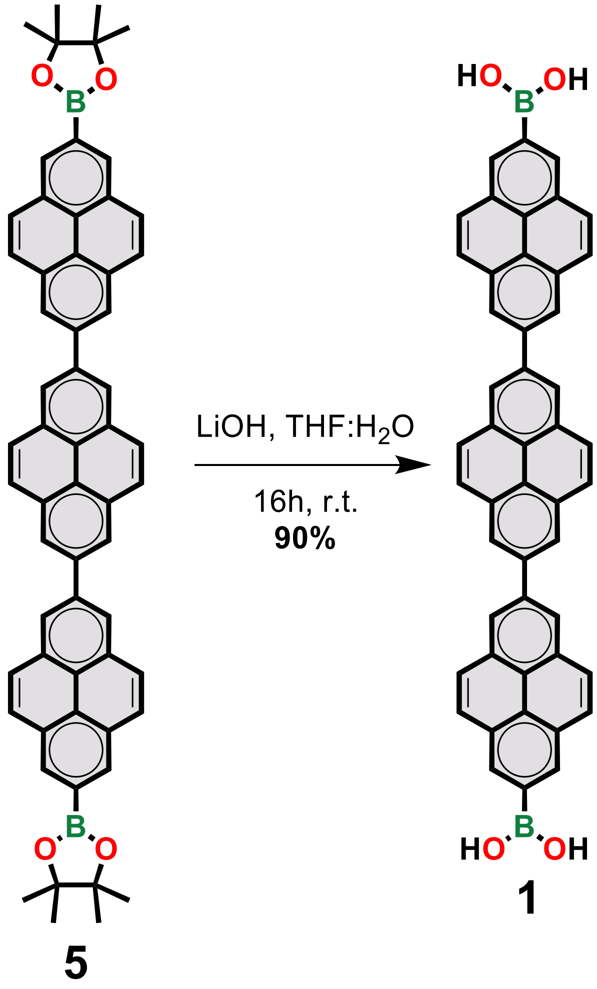

To a solution of 7,7''-bis(4,4,5,5-tetramethyl-1,3,2-dioxaborolan-2-yl)-2,2':7',2''-terpyrene (**5**) (50 mg, 0.059 mmol) in THF (5 mL), an aqueous solution of lithium hydroxyde (LiOH) (250 mg, 5mL, 10.4 mmol) was added dropwise. It was stirred for 16 h at r.t. Organic solvents were removed and hydrochloric acid was added (conc., 4 mL) and precipitate was formed. It was filtered, washed with water (30 mL), methanol (30 mL), diethyl ether (30 mL) and chloroform (30 mL) to give an off-white solid (36.6 mg, 90%).

^{1}H NMR (500 MHz, DMSO-d_6) δ 9.03 (s, 4H), 8.99 (s, 4H), 8.76 (s, 4H), 8.48 (s, 4H), 8.46 (s, 4H), 8.38 (d, *J* = 9.1 Hz, 4H), 8.29 (d, *J* = 9.1 Hz, 4H).

^{13}C NMR (126 MHz, DMSO-d_6) δ 138.90, 132.26, 131.96, 131.82, 130.25, 128.86, 128.82, 128.68, 127.81, 125.29, 125.05, 124.99, 124.44, 123.77, 123.62.

MS (MALDI-TOF) (m/z): calculated for $C_{48}H_{28}B_2O_4$: 690.217; found: 690.215 $[M]^+$

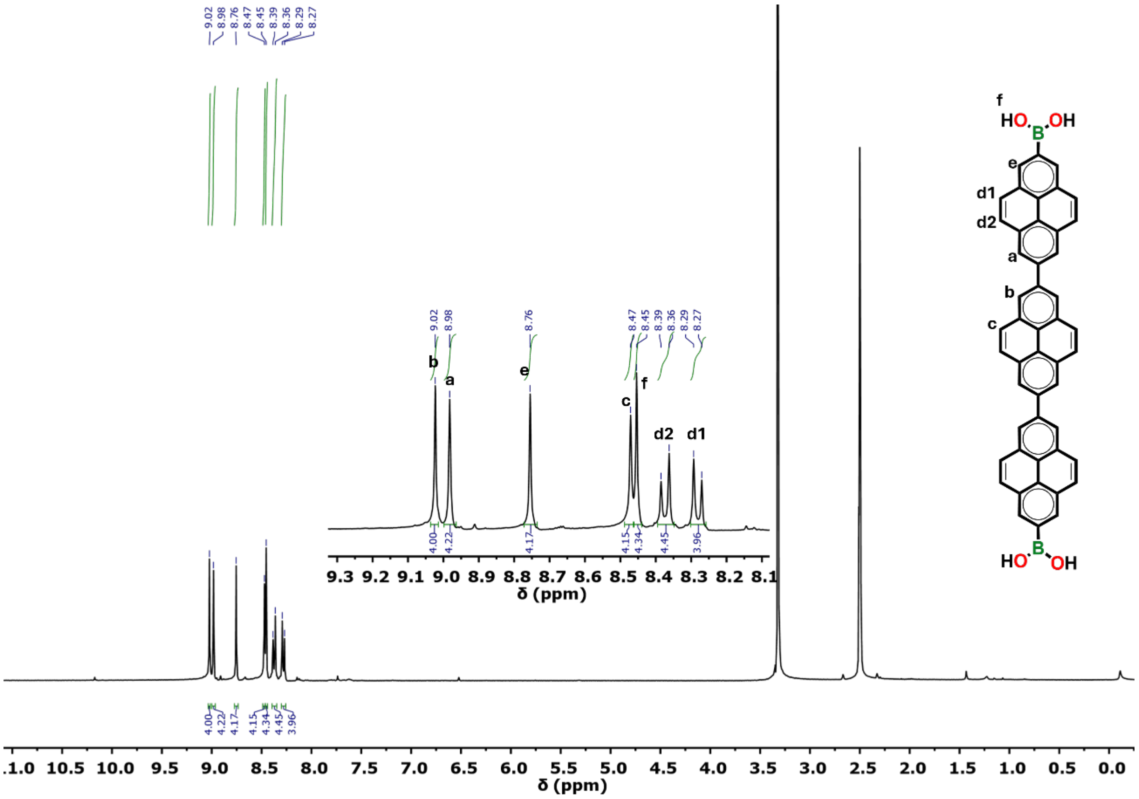

Figure S29. ^{1}H NMR spectrum of compound **1**.

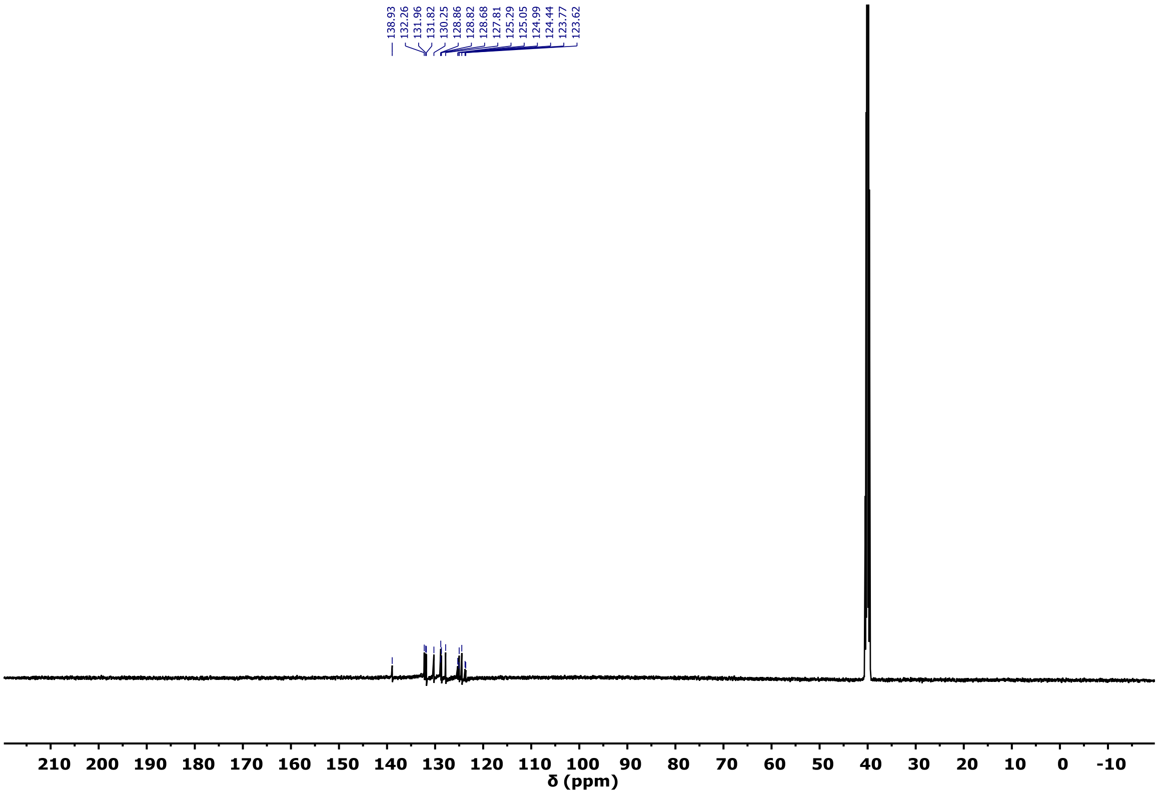

Figure S30. ^{13}C NMR spectrum of compound **1**.

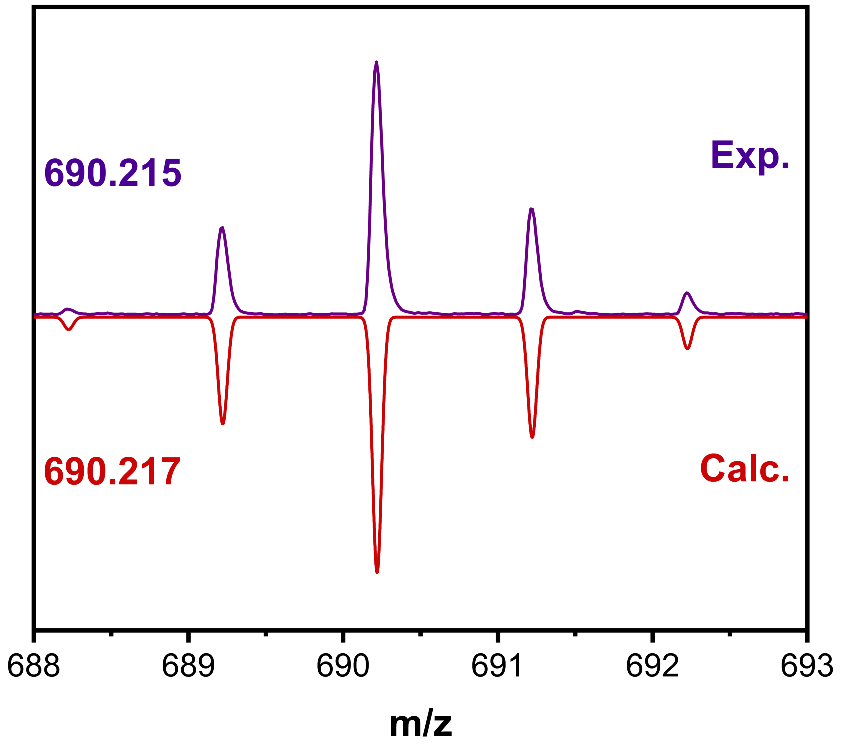

Figure S31. MS spectrum of compound **1**.

Joa-COF-1

2,3,10,11,18,19-hexamethoxy-cata-hexabenzocoronene (HBC) (13 mg 0.0187 mmol) and diboronicterpyrenyl acid (20,1 mg, 0,028 mmol) were placed in a pre-scored 5 mL ampoule and 2 mL of mesitylene:dioxane (1:2, *v:v*) were added under nitrogen. After 3 freeze-pump-thaw cycles, the ampoule was closed using a flame. The ampoule was heated at 120 °C for 72h. It was filtered and washed with dry THF (50 mL), dry acetone (50 mL) and dry hexane (30 mL). **Joa-COF-1** was obtained as a greedish solid (22 mg, 80%).

SS^{1}H-NMR (δ) (ppm): 6.39.

SS^{13}C-NMR (δ) (ppm): δ 144.33, 138.34, 125.94, 108.75.

ATR-FTIR (cm^{-1}): 1624, 1608, 1568, 1426, 1352, 1317, 1232.

References

[1] M. Martínez-Abadía, C. T. Stoppiello, K. Strutynski, B. Lerma-Berlanga, C. Martí-Gastaldo, A. Saeki, M. Melle-Franco, A. N. Khlobystov, A. Mateo-Alonso, *J. Am. Chem. Soc.* **2019**, *141*, 14403-14410.

[2] A. G. Crawford, Z. Liu, I. A. Mkhalid, M. H. Thibault, N. Schwarz, G. Alcaraz, A. Steffen, J. C. Collings, A. S. Batsanov, J. A. Howard, T. B. Marder, *Chem. Eur. J.* **2012**, *18*, 5022-5035.

[3] E. Caldeweyher, C. Bannwarth, S. Grimme, *J. Chem. Phys.* **2017**, *147*, 034112.

[4] E. Caldeweyher, S. Ehlert, A. Hansen, H. Neugebauer, S. Spicher, C. Bannwarth, S. Grimme, *J. Chem. Phys.* **2019**, *150*, 154122.

[5] E. Caldeweyher, J. M. Mewes, S. Ehlert, S. Grimme, *Phys. Chem. Chem. Phys.* **2020**, *22*, 8499-8512.

[6] B. Aradi, B. Hourahine, T. Frauenheim, *J. Phys. Chem. A* **2007**, *111*, 5678-5684.

[7] J. P. Perdew, K. Burke, M. Ernzerhof, *Phys. Rev. Lett.* **1996**, *77*, 3865-3868.

[8] J. P. Perdew, K. Burke, M. Ernzerhof, *Phys. Rev. Lett.* **1997**, *78*, 1396–1396.

[9] A. Ambrosetti, A. M. Reilly, R. A. DiStasio, Jr., A. Tkatchenko, *J. Chem. Phys.* **2014**, *140*, 18A508.

[10] A. Tkatchenko, R. A. DiStasio, Jr., R. Car, M. Scheffler, *Phys. Rev. Lett.* **2012**, *108*, 236402.

[11] V. Blum, R. Gehrke, F. Hanke, P. Havu, V. Havu, X. Ren, K. Reuter, M. Scheffler, *Comput. Phys. Commun.* **2009**, *180*, 2175-2196.

[12] A. Marek, V. Blum, R. Johanni, V. Havu, B. Lang, T. Auckenthaler, A. Heinecke, H. J. Bungartz, H. Lederer, *J. Phys. Condens. Matter* **2014**, *26*, 213201.

[13] V. W.-z. Yu, F. Corsetti, A. García, W. P. Huhn, M. Jacquelin, W. Jia, B. Lange, L. Lin, J. Lu, W. Mi, A. Seifitokaldani, Á. Vázquez-Mayagoitia, C. Yang, H. Yang, V. Blum, *Comput. Phys. Commun.* **2018**, *222*, 267-285.

[14] L. Sarkisov, A. Harrison, *Mol. Simul.* **2011**, *37*, 1248-1257.

[15] L. Sarkisov, R. Bueno-Perez, M. Sutharson, D. Fairen-Jimenez, *Chem. Mater.* **2020**, *32*, 9849-9867.
